# Sugar-Coded Signals: Glycan-Based Biomarkers in Rheumatoid Arthritis

**DOI:** 10.1016/j.mcpro.2026.101633

**Published:** 2026-08-04

**Authors:** Michal Hires, Stefania Hroncekova, Viera Dujnič, Veronika Solovicova, Veronika Pinkova Gajdosova, Eduard Jane, Lenka Lorencova, Tomas Bertok, Alica Vikartovska, Anna Datkova, Peter Kasak, Radek Kucera, Jan Tkac

**Affiliations:** 1 Institute of Chemistry, Slovak Academy of Sciences, Bratislava, Slovakia; 2 Centre for Advanced Materials, Qatar University, Doha, Qatar; 3 Department of Pharmacology and Toxicology, Faculty of Medicine in Pilsen, Charles University, Pilsen, Czech Republic; 4 Department of Immunochemistry Diagnostics, University Hospital and Faculty of Medicine in Pilsen, Charles University, Pilsen, Czech Republic

**Keywords:** rheumatoid arthritis (RA), biomarker discovery, glycans, lectins, immunoglobulins, seropositive RA, seronegative RA

## Abstract

This review paper summarizes the pathophysiology of rheumatoid arthritis (RA), its incidence and etiology, and conventional RA protein-based biomarkers. A brief overview of novel protein-based biomarkers and biomarkers with potential to be used in the diagnostics of seronegative RA is also provided. The core of the review resides in the comprehensive characterization of the glycosylation of immunoglobulins and their utility as novel biomarkers for the diagnostics of RA and seropositive RA. Two main approaches to the analysis of glycans present on immunoglobulins and other proteins are provided, including instrument-based and lectin-based approaches showing their clinical performance as RA biomarkers.

## Highlights

• Pathophysiology of rheumatoid arthritis (RA), its incidence/etiology is provided.
• The article shortly describes conventional RA biomarkers.
• A brief overview of novel protein-based RA biomarkers and biomarkers is given.
• Glycosylation of immunoglobulins (IgGs) as novel RA biomarkers is provided.
• Two approaches to the analysis of IgG’s glycans & other proteins are discussed.

## Graphical Abstract

**Figure:**
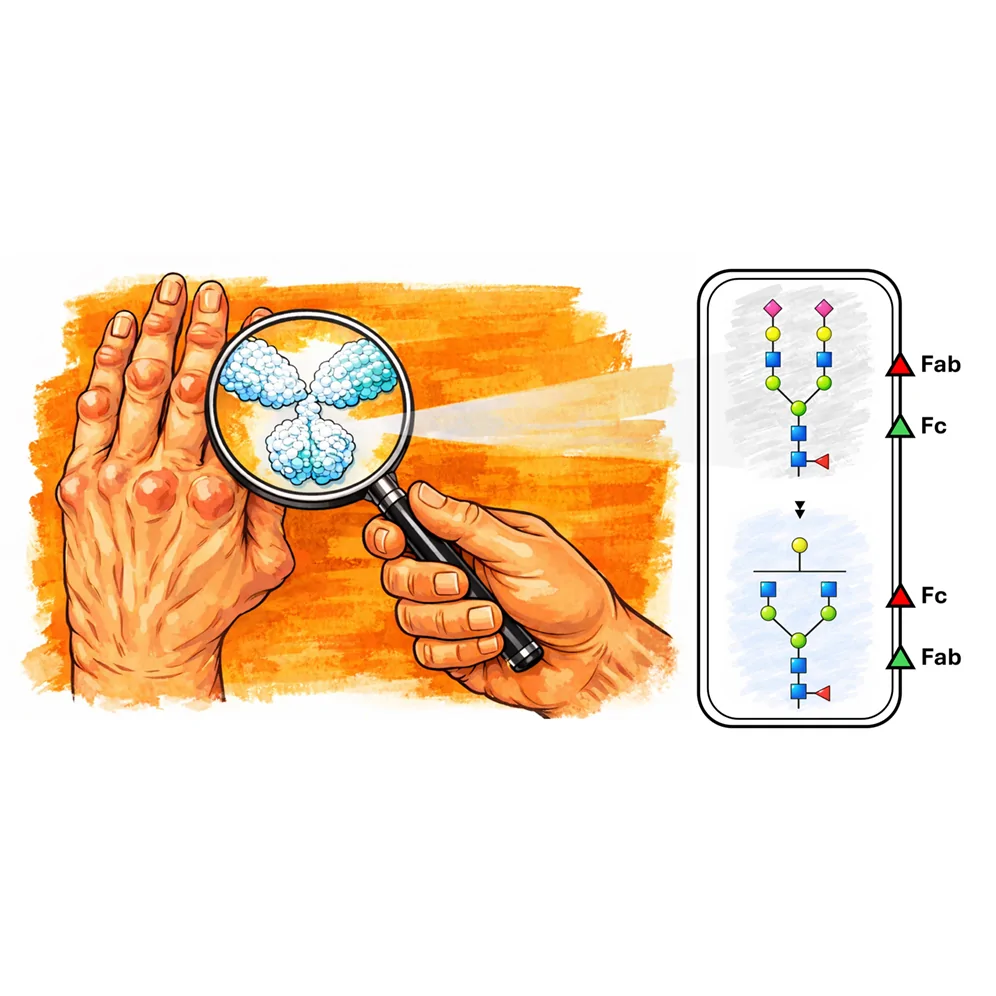

## In Brief

This review paper summarizes the pathophysiology of rheumatoid arthritis (RA), its incidence and etiology, conventional and novel RA protein-based biomarkers for diagnostics of RA and seronegative RA. A comprehensive characterization of the glycosylation of immunoglobulins and their utility as novel biomarkers for the diagnosis of RA and seropositive RA is provided as well. Analysis of glycans present on immunoglobulins and other proteins, including instrument-based and lectin-based approaches, is discussed, showing their clinical performance as RA biomarkers.

This review focuses on the description and clinical performance of potential serological rheumatoid arthritis (RA) biomarkers, including autoantibodies, proteins, and glycoproteins. The main reason behind this selection is that liquid biopsy-based approaches and, in particular, serological RA biomarkers may offer noninvasive alternative assay formats to profile synovial tissues, thereby improving accessibility and enabling dynamic monitoring of disease activity (1). The reader is advised to read other excellent review papers to gain further details of other types of RA biomarkers, including genetic, epigenetic, imaging, metabolic and microbiota-based biomarkers (1, 2, 3, 4, 5). Research published in 2025 and summarized in a short article provides new insights into the preclinical phase preceding the RA onset, encompassing dietary risk factors, immunological events, and potential ways to prevent progression of the RA disease (6).

To bridge the gap between routine clinical practice and basic clinical research, greater emphasis should be placed on technology translation. Thus, rigorous cost-effectiveness analyses are required to demonstrate the clinical utility of biomarkers (1). The assay should be performed to allow high assay throughput in multiplexed assay formats and to be compatible with instrumentation used in standard clinical practice. There are other issues meriting attention when identifying new biomarkers and novel assay concepts, that is, cost per sample, regulatory complexity behind assay validation, and the requirement to have highly trained operators/clinicians for performing the assays and for proper and robust data evaluation.

This is the first review paper focusing on glycans as novel RA biomarkers, discussing their clinical parameters and different approaches to glycan analysis. In addition, we discuss other protein-based novel RA biomarkers, especially applicable to the diagnosis of the seronegative form of RA, which is problematic to diagnose using standard serological biomarkers.

## RA Pathophysiology

RA as a systemic, slowly progressing autoimmune inflammatory disease starting with arthralgia as a key preclinical manifestation. RA affects especially joints and periarticular soft tissues with a wide range of symptoms, some of which are not specific (7). RA starts in the joints of the hands, feet and wrists, but also affects other large joints in the body (8, 9). If the disease is not treated, it can end in permanent joint damage with a loss of function.

RA is characterized by heterogeneous clinical symptoms and often unpredictable disease development, which complicates early diagnostics and the implementation of personalized therapeutic strategies (1). A recent review paper stated that diagnostic criteria for RA did not exist (10). Furthermore, it was claimed that, due to the fact that clinically significant biomarkers for diagnostics, prognosis, and therapy monitoring had yet to be identified, precision medicine remained an elusive concept in rheumatology (2). This is supported by the fact that ∼50% of RA patients fail to respond to the prescribed therapy adequately and >60% of RA patients require at least 3 treatment changes to reduce disease activity and/or achieve disease remission (3). Hence, the diagnosis of RA is complex, requiring qualified and experienced medical personnel.

RA patients have painful and/or swollen joints and changes in blood components, including increased CRP (C-reactive protein) and changes in ESR (erythrocyte sedimentation rate) due to ongoing inflammation (11). A certain distinguishing feature of the RA disease is morning stiffness of the joints, mostly symmetrical damage as well as fatigue after even light exertion.

The main feature of the RA disease is the serological presence of autoantibodies recognizing the Fc domain of the body’s own immunoglobulins G (IgGs) antibodies, that is, RF (rheumatoid factor), anti-citrullinated protein antibodies (ACPA) and others (7). RA patients who have these autoantibodies are classified as being *seropositive* (SP) RA patients, whereas individuals who exhibit clinical features of RA in the absence of these autoantibodies are defined as being *seronegative* (SN) RA patients (∼20–30% of RA cases) (12). The heritability of SP RA and SN RA is observably different, 40% to 65% and 20%, respectively (13, 14). Details of the types of cells involved and genetic differences between SP and SN RA cohorts are provided in the recent review paper (12).

The autoimmune response of RA patients is characterized by the blood-circulating ACPA, which may be detectable several years *prior* to RA onset. ACPA-positive, i.e. SP RA patients have a more severe disease progression and are less likely to achieve remission than SN RA patients (15). ACPA responses are dynamic during the progression towards RA, with increasing antibody levels and expansion of the range of epitopes recognized by such antibodies. However, the levels of autoantibodies are not related to a long-term treatment response and do not predict attainment of drug-free remission (15). Importantly, glycoforms of total IgGs, rather than total ACPA IgG levels, have shown significant correlations with inflammatory markers such as ESR and DAS28 (disease activity score examined using 28 joints), and enabled the stratification of patients into distinct groups according to disease status (16).

SN RA represents a distinct clinical entity characterized by polyarthritis and incompletely defined pathogenic mechanisms when compared to SP RA. The clinical course of SN RA is generally associated with less severe joint destruction; however, therapeutic strategies largely parallel those used for SP RA (17). Furthermore, since SN RA patients do not have serological biomarkers, these patients are diagnosed at a later stage with a higher level of inflammation than the SP RA patients (12). In addition, epidemiological studies have demonstrated an increasing incidence of SN RA when compared to the previous decades (7).

RA represents a significant socioeconomic problem. Individuals with RA experience a decreased quality of life associated with joint damage. Chronic inflammation exhausts the patient and spreads to other parts of the body, such as the kidneys, skin (rashes, nodules), eyes (dryness, inflammation), lungs (risk of pleurisy, formation of lung nodules), etc. (9, 18, 19). RA patients face a significant risk of cardiovascular problems due to systemic inflammation (20). Inflammation of blood vessels can lead to damage and increased blood clot formation (21). Major complications include a direct link to an increased risk of heart attack, heart failure, or stroke (22). Furthermore, RA patients require increased healthcare costs and impose a significant economic burden on society, associated with reduced work performance. The burden of RA is significant, with approximately 33% of patients forced to discontinue employment within 2 years of RA onset. Moreover, the economic impact of RA is considerable; in the UK, the annual cost of RA, including sick leave and disability, was estimated at £ 3.8 to 4.8 billion in 2009 (12). Expenditures related to the treatment of osteoarthritis and RA increased to £ 10.2 billion in 2017, while the combined annual costs associated with sick leave attributable to RA and osteoarthritis were estimated to reach £ 100 billion in 2019 (12). On the other hand, advances in RA treatment are associated with a decline in the incidence of several comorbidities; however, lung diseases, anxiety and depression remain prevalent among RA patients (23).

Several scoring systems are used to assess disease activity in RA, but DAS28 is the most commonly employed. This composite index takes into account tenderness and swelling in 28 joints (24).

## Incidence and Etiology

RA, with an incidence of 0.5 to 1%, belongs to the most frequent chronic inflammatory diseases (12), affecting 17.5 million people worldwide in 2020, with prevalence 2.45-times greater in females (25). Sex and gender differences in RA are discussed in a recent review paper (26). It is estimated that there will be 31.7 million RA patients worldwide by 2050 (8). Further risk factors are summarized in Table 1 (27).

**Table 1:** Risk of Development of RA Within 1 year (Data Taken from Open Access Publication (27))

Population	Estimated risk (%)
A whole population	∼0.04
Individuals, who are the 1st-degree relatives to RA patients	∼0.2
ACPA-negative patients having musculoskeletal pain	∼0.8
ACPA-positive patients in a whole population	∼2.9
ACPA-and RF-negative patients with clinically suspect arthralgia	∼8
ACPA-positive patients having musculoskeletal pain	∼15–42
ACPA-and RF-positive patients with clinically suspect arthralgia	∼57

The exact cause of the disease is unknown; however, the etiology is heterogeneous and influenced by environmental factors, genetic predisposition, epigenetic modifications (i.e. histone acetylation, DNA methylation, phosphorylation, ubiquitylation, and sumoylation), and post-translational modifications (1, 7). It is believed to be a combination of several factors. A positive family history enhances the risk of the disease by 2 to 5 times when compared with the whole population (7). In people with a predisposition to RA, risk factors including exposure to dust and viruses, smoking (the strongest environmental risk factor), dietary habits, obesity, low socio-economic status and changes in the lungs, gut, and oral microbiome all contribute to the breakdown of tolerance responsible for the production of inflammatory cytokines and autoantibodies and ultimately for RA initiation (7, 28, 29). Such breaks may be induced by the introduction of *N*-linked glycosylation sites within the antigen-binding pocket of the B-cell receptor selectively, leading to structural alterations of the binding site and enhanced B-cell activation (17). Alternatively, ACPA harboring variable domain glycans might be involved in a break of tolerance (30).

**Fig. 1:**
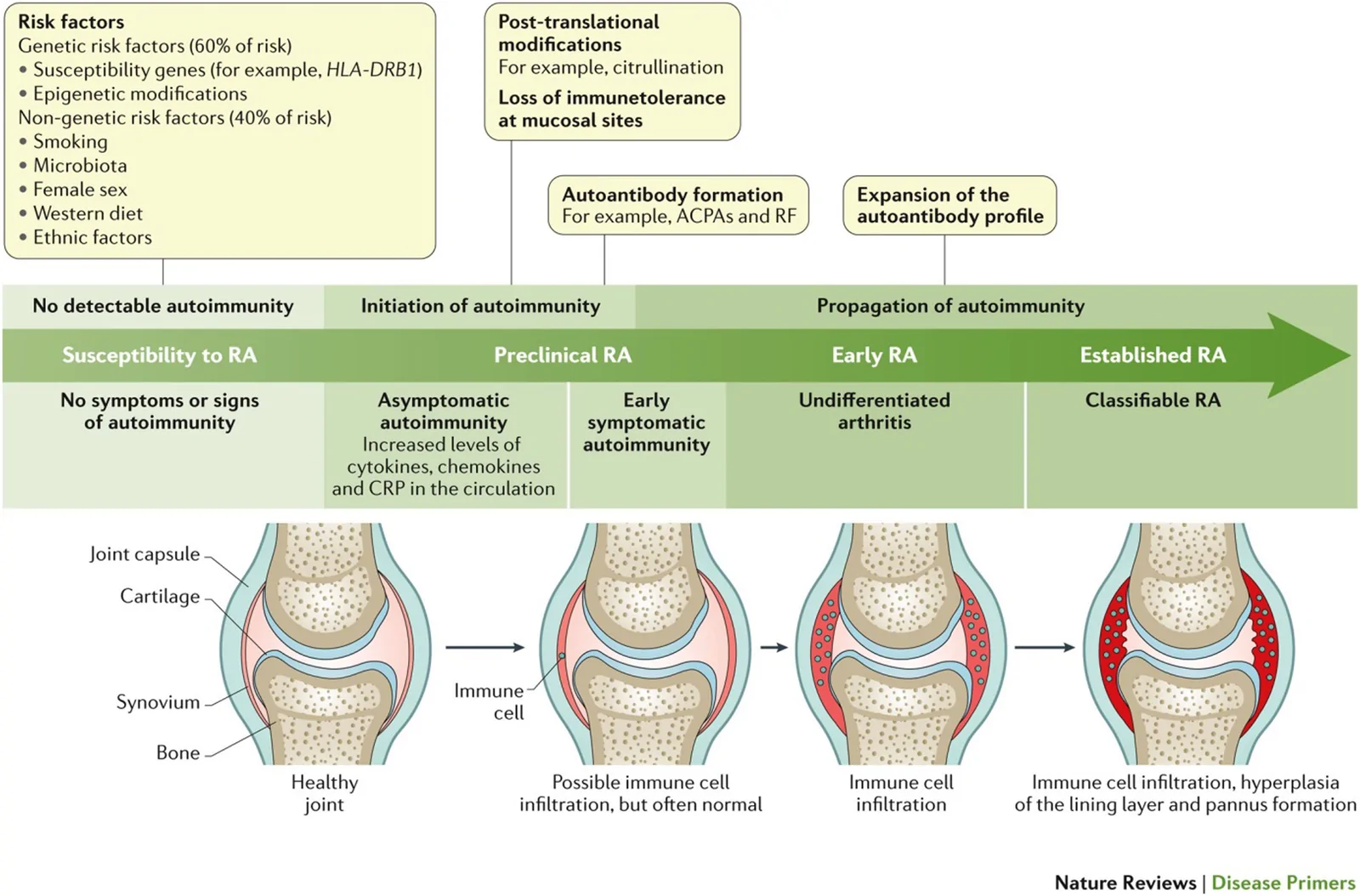
**RA development and progression.** There are genetic and other risk factors contributing to RA, with several of them important to reach a threshold triggering RA. RA progression is associated with an immune response against modified self-proteins. ACPA: anti-citrullinated protein antibodies; CRP: C-reactive protein; RF: rheumatoid factor. Reproduced with permission from Springer Nature from ref. (31).

The preclinical stage of RA starts on mucosal surfaces present in the lungs, mouth and gastrointestinal tract with a local and systemic production of autoantibodies (17). Such autoantibodies are present in the blood 4.5 years prior to the onset of the disease (17). An increased level of autoantibodies, an increased number of proteins recognized by autoantibodies, along with an enhanced pool of pro-inflammatory proteins in blood result in joint inflammation (Fig. 1) (17, 31).

Genetic predisposition to RA is the strongest risk factor, with heritability estimates close to 60% (1). Modern approaches have identified at least a hundred alleles linked to RA risk, most of which are associated with immune mechanisms, with some of them being common to other chronic inflammatory conditions (32). The most important is the HLA (human leukocyte antigen) system and especially the HLA-DRβ1 gene (17). There is a well-characterized sequence (amino acids 70–74) in the hypervariable domain of the HLA-DRβ1 chain, known as the shared epitope, linked to an increased risk of RA, but also to disease severity, which also influences treatment outcomes for some of the biologics. The relatively low concordance of RA in monozygotic twins (∼15%) suggests that epigenetic factors in combination with environmental factors are also very important (17). Thus, genetic factors are behind host susceptibility, but epigenetic mechanisms affecting gene expression contribute to RA heterogeneity, dysregulation of the immune system and variability in therapeutic outcome (28).

The phenomenon of the interaction of the microbiome with the human body is experiencing a great expansion; the observation is that the influence of microorganisms on the onset and progression of many, especially autoimmune, diseases is greater than expected. Data obtained in animal models of arthritis indicate a key role of the gut microbiome in the development of the disease (28). Initial studies in humans have identified gastrointestinal dysbiosis in RA. Observations also suggest an association of RA with periodontal disease, although the exact mechanism is unknown. One hypothesis assumes that *Porphyromonas gingivalis,* a bacterium frequently found in periodontitis, promotes aberrant citrullination and thereby contributes to a local impairment of tolerance to citrullinated peptides. Other infectious agents (i.e. *Proteus mirabilis*, *Escherichia coli*, *Mycoplasma* sp. and *Epstein–Barr* virus) are also thought to be associated with the development of RA, particularly via molecular mimicry (28). A detailed overview discussing gut microbiota in relation to RA is discussed in a recent review paper (3).

## RA Biomarkers

Below, we briefly discuss conventional RA biomarkers used in the management of RA patients and novel autoantibody or protein-based biomarkers. Their clinical performance is summarized in Table 2, Table 3, Table 4. Here we also discuss the potential of novel approaches towards diagnosing RA based either on analysis of multiple biomarkers or using advanced computation methods. We provide evidence that exosomes, as small extracellular vesicles, can be a precious source of RA protein-based biomarkers.

**Table 2:** Summary of Autoantibody-Based RA Biomarkers (updated from Refs. (1, 12, 37))

Biomarkers	Clinical parameters	Target	Comments
RF	Sens.: 60–90% Spec.: 50–90%	Fc domain of IgGs	Diagnostic & prognostic SP RA biomarker, prediction of treatment
ACPA	Sens.: 60–70% Spec.: 89–95%	Citrullinated proteins	Diagnostic & prognostic SP RA biomarker, prediction of treatment
anti-CarP	Sens.: 30–50% Spec.: ∼90%	Carbamylated proteins	- Prior to RA onset - Also as prognostic biomarkers - Diagnostics of SN RA
anti-PAD	Found in 35% of RA patients Sens.:20–45% Spec.: >90% for anti-PAD4	Peptidyl-arginine deaminase enzymes	- Anti-PAD2 Abs linked to a milder RA form - Anti-PAD3 & Anti-PAD4 Abs linked to more severe RA - Anti-PAD3 Abs present before RA onset
AMA	-	Extracellular mitochondria	- To predict severe RA for both SP and SN RA forms - Anti-mitofusin-1 Abs as SN RA biomarker for patients with severe RA
Anti-RA33	Sens.: 32% Spec.: 90%	RA33 - a nuclear protein associated with splicing of mRNA	Biomarker to evaluate risk for inflammatory arthritis
Anti-SA Abs	Sens.: 20–30% Spec.: >95%	Citrullinated vimentin	Prediction of disease severity and joint erosions
Anti-mutated citrullinated vimentin Abs	Sens.: 70–80% Spec.: 85–95%	Mutated citrullinated vimentin	SN RA biomarker to evaluate RA activity/severity
Anti-AGE and MAA	-	Glycation end-products and malonidaldehyde–acetaldehyde adducts	Biomarker to reveal radiological progression in SN RA patients

ACPA, anti-citrullinated protein antibodies; AMA, anti-mitochondrial antibodies; Anti-AGE, antibodies against advanced glycation end-products; MAA, antibodies against malondialdehyde–acetaldehyde adduct-modified proteins; anti-carP, anti-carbamylated protein antibodies; anti-PAD, antibodies against peptidylarginine deiminase; Anti-RA33, antibodies against heterogeneous nuclear ribonucleoprotein (hnRNP) A2/B1, a protein involved in mRNA splicing; Anti-SA Abs, antibodies against citrullinated vimentin; RF, rheumatoid factor; Sens., sensitivity; SN RA, seronegative rheumatoid arthritis; SP RA, seropositive rheumatoid arthritis; Spec., specificity.

**Table 3:** Summary of Inflammatory-Based RA Biomarkers (taken from Ref. (1))

Biomarkers	Clinical parameters	Presence in other conditions	Comments
CRP	Sens.: 40–60% Spec.: ∼40%	Increased level associated with several inflammatory, infectious, and tissue-damaging processes	Part of DAS, applicable for RA monitoring and treatment outcome
ESR	Sens.: 40–60% Spec.: ∼40%	Enhanced level linked to several inflammatory and infectious diseases, including also oncological ones	Part of DAS, applicable for RA monitoring and treatment outcome
Amyloid A	Sens.: 40–60% Spec.: 40–60%	Increased level associated with several inflammatory diseases	Applicable for RA monitoring disease activity and as prognostic biomarker
MMP-3	Sens.: 40–80% Spec.: 50–70%	Biomarker for other autoimmune diseases	A biomarker applicable for destruction of joints and cartilage
Calprotectin	Sens.: 60–80% Spec.: ∼90%	Biomarker for other autoimmune diseases and infections	Linked to RA disease activity; predicting poor radiographic results
14-3-3-η protein	Sens.: 60–80% Spec.: 80–90%	Osteoporosis, other autoimmune diseases	Diagnostic and prognostic biomarker and marker of RA severity; applicable especially for severe RA disease
TNF-α	Sens.: 40–70% Spec.: 40–60%	Various autoimmune/inflammatory diseases	Key therapeutic target, monitoring disease activity
IL-6	Sens.: 40–70% Spec.: 40–60%	Several autoimmune and inflammatory conditions	Therapeutic biomarker, and for monitoring of RA activity

14-3-3-η, a specific isoform of the 14-3-3 family of chaperones; CRP, C reactive protein; ESR, erythrocyte sedimentation rate; IL-6, interleukin 6; MMP-3, matrix metallo-proteinase 3; Sens., sensitivity; Spec., specificity; TNF-α, tumour necrosis factor α

**Table 4:** Novel protein-based RA biomarkers

Biomarkers	Clinical parameters	Samples	Refs.
LRG1	AUC = 0.795 (RA *versus* HI)	78 RA + 20 HI	(73)
OPN	-	Meta-analysis	(75)
Anti-CSP	AUC = 0.78–0.88 (RA *versus* HI)	4 different cohorts	(78)
Anti-CSP + ACPA	AUC = 0.85–0.97 (RA *versus* HI)	4 different cohorts	(78)
Galectin 1	AUC = 0.76–0.95 (RA *versus* HI)	Several cohorts	(66, 67, 68, 69)
Galectin 3	AUC = 0.88 (RA *versus* HI)	Two cohorts	(69)
Galectin 4	Sens.: 93%; spec.: 79% (RA *versus* HI)	60 RA + 30 HI	(68)
Tenascin C	Sens.: 97%; spec.: 100% (RA *versus* HI)	60 RA + 30 HI	(80)
Tenascin C	Sens.: 93%; spec.: 100% (SN RA *versus* HI)	30 SN RA *versus* 30 HI	(80)
Metalloproteinase 3	AUC = 0.93 (SN RA *versus* HI)	145 SN RA + 38 HI	(81)
Metalloproteinase 3	AUC = 0.92 (RA *versus.* HI)	290 RA + 38 HI	(81)
Syndecan-1	Sens.: 84%; spec.: 52%	75 RA + 25 HI	(88)

ACPA, anti citrullinated protein antibodies; Anti-CSP, anti-citrullinated scavenger receptor A peptide antibody; AUC, area under curve, i.e. receiver operating curve; HI, healthy individuals; LRG1, leucine-rich α-2-glycoprotein 1; OPN, osteopontin; RA, rheumatoid arthritis; Sens., sensitivity; SN RA, seronegative rheumatoid arthritis; Spec, specificity.

### SP RA Biomarkers

Two biomarkers are used in the classification of SP RA—that is, rheumatoid factor (RF) and ACPA. RA patients having autoantibodies are clinically distinct from SN RA individuals, exhibiting a more severe RA development and progression, and a higher prevalence of extra-articular manifestations than are observed in SN RA patients (30). These autoantibodies are characteristic of RA and are present in 50 to 70% of RA patients at diagnosis; they are remarkably stable throughout the disease course. In SP RA patients, the effective treatment is associated with a reduction in autoantibody levels. This decline is more pronounced and more frequently observed for RF, in some cases resulting in reversion to RF negativity. By contrast, the levels of ACPA rarely fall below the detection threshold, and patients typically do not become ACPA-negative (33). The clinical parameters and characteristics of various types of RA biomarkers are summarized in Table 2, Table 3, Table 4.

#### Rheumatoid Factor (RF)

RF was first described in the RA patient’s serum in 1937 (34). Unlike most other autoantibodies, RF predominantly occurs as immunoglobulin M (IgM), while IgG and IgA subtypes are detected less often.

RF recognizes conformational epitopes within the Fc region of IgG, particularly when structural alterations increase the accessibility of these epitopes. It is well known that glycans within the Fc domain of IGs show noncovalent interactions with the Fc Cγ2 domain of IgG (35). Although IgG glycosylation does not directly constitute an antigenic determinant for RF binding, it plays a crucial role in maintaining the conformation and dynamics of the IgG Fc domain and may therefore modulate RF–IgG interactions.

RFs of all immunoglobulin types may be present in blood at early stages of RA and even before the clinical onset of the disease by several years. Notably, IgA-RF has a prognostic relevance, since it is associated with a greater disease severity, including joint erosion, progressive disease course, and extra-articular manifestations (30).

RF is present in blood at typical values of 0 to 20 IU/ml. RF is not RA-specific since elevated values are detected only in ∼60 to 80% of patients with RA and about 5% of positive tests are observed in people without RA (28). The literature reports considerable variability in the clinical performance of RF for RA, with sensitivity in the range of 60 to 90% and specificity in the range of 50 to 90% (Table 2) (1, 12, 36, 37). This might be because RF is present in other diseases, including autoimmune ones and chronic infections but can be present in healthy individuals (HI, especially the elderly) (28).

#### ACPA

The limited specificity of RF prompted efforts in the latter half of the 20^th^ century to identify more disease-specific autoantibodies, ultimately leading to the discovery of ACPA in 1964 (36) and to the development of more targeted diagnostic assays based on analysis of ACPA in the 1990s (12). ACPA concentration of less than 20 IU/ml is considered normal and is detectable in 60 to 70% of RA patients (30). ACPAs as RA biomarkers exhibit excellent specificity (89–95%) and moderate sensitivity (∼41–80%) to RA (Table 2) (28, 36).

**Fig. 2:**
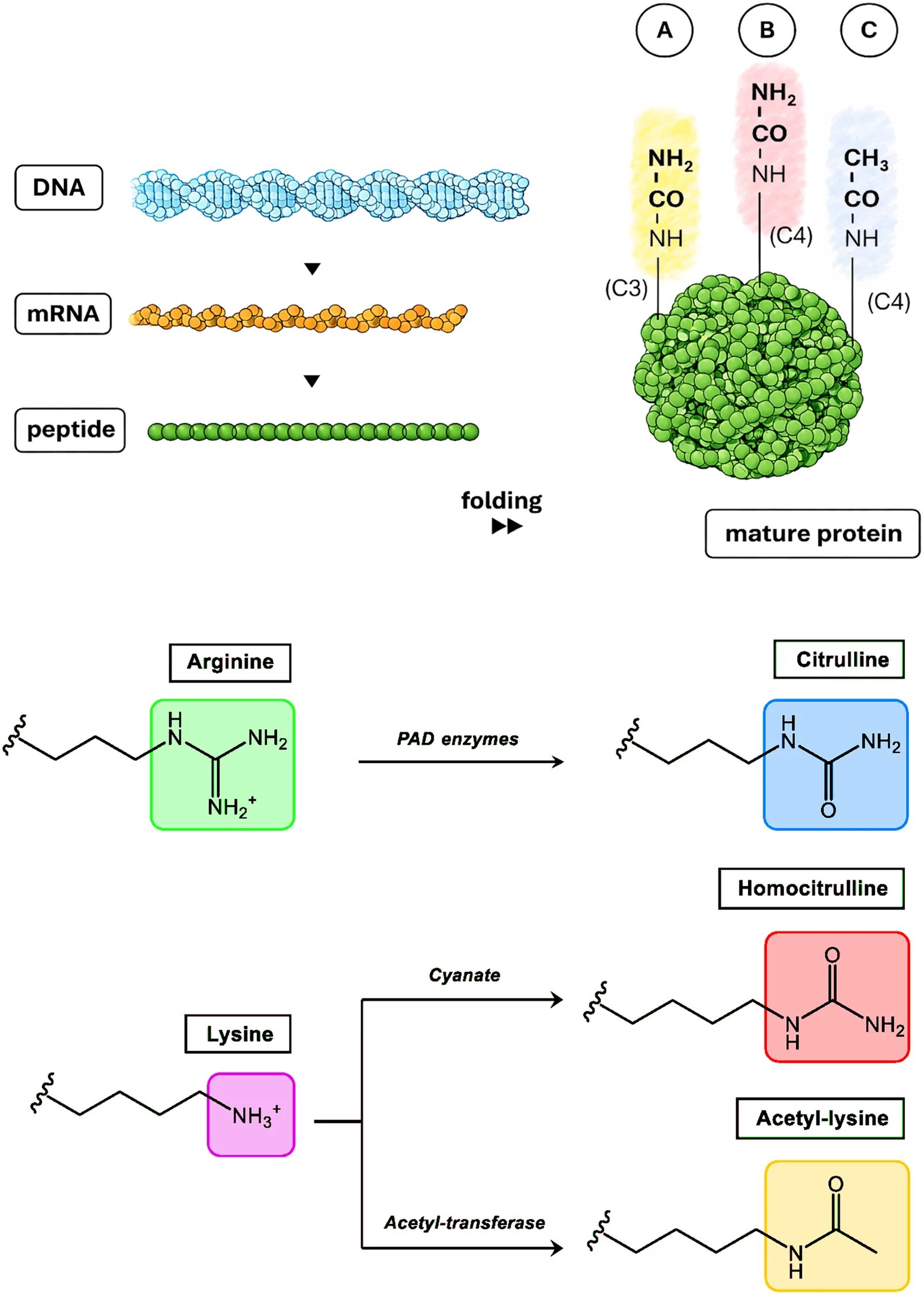
**The mature proteins undergoing chemical or enzymatic transformation are closely linked to RA.** The majority of RA patients (50–70%) have antibodies against modified proteins containing (*A*) citrulline, (*B*) homocitrulline, and/or (*C*) acetyllysine. C3 refers to –(CH_2_)_3_ C4 refers to –(CH_2_)_4_. Amended based on Ref. (30) with permission. The lower part of the figure shows chemical modifications of amino acids. Taken from an open-access publication (38).

Citrullination is modification of mature already synthesized proteins by enzymatic conversion of arginine to citrulline (Fig. 2) (30, 38). This deimination reaction is catalyzed by peptidylarginine deiminase (PAD) enzymes and is distinct from citrulline generation in the urea cycle or through pathways involving the nitric oxide synthase family.

Citrullination is a normal physiological process with multiple functions in the human body. The enzymatic transformation of arginine to citrulline removes a positive charge and thus enhances hydrophobicity, which can alter protein folding and, consequently, affect protein structure and function, including molecular interactions and enzymatic activity (39). The best-characterized antigens recognized by ACPA include citrullinated filaggrin, type II collagen (CII), α-enolase, fibrinogen, vimentin, etc. (38, 40).

Citrullination of proteins is elevated after smoking, since tobacco increases PAD expression. This is one of the reasons why smoking is attributed to the onset and worsening of autoimmune diseases. Citrullination is a natural cellular process involved in gene expression, cell differentiation, apoptosis (programmed cell death), skin formation and immunity. Dysregulation of the process leads to the development of several diseases, including autoimmune and neurodegenerative ones (30).

### SN RA and Novel Biomarkers

It is estimated that at least 20 to 30% of RA patients are SN RA patients (12). In the groups of RA patients, new autoantibodies, including anti-CarP antibodies (*i.e*. antibodies to carbamylated proteins) and anti-PAD4 antibodies, may represent promising biomarkers, especially for SN RA patients. In the future, these biomarkers can be used for RA diagnostics and disease activity monitoring for proper stratification of RA patients (28). A recent paper suggests analysis of the level of 14-3-3-η protein (i.e. a specific isoform of the 14-3-3 family of chaperones) and anti-CarP for diagnostics of SN RA patients and determination of calprotectin level for monitoring of RA patients and efficiency of RA treatment (Tables 2 and 3) (1, 37).

SP RA patients have the presence of RF and/or ACPA in the blood, and such patients are considered to be a distinct group of patients when compared to SN RA patients. Although smoking is generally a strong risk factor for RA in general, it is a strong risk factor mainly for the SP RA subtype. The SN RA subtype is especially linked to hormone-related risk factors (41).

Array-based assay formats have identified novel SN RA biomarkers (i.e. antibodies targeting peptides released from joints). Such studies also showed that 20 to 30% of SN RA patients have autoantibodies targeting not only native proteins or unmodified peptides but also modified peptides (42). Thus, we can assume that there are at least three subcategories of SN RA patients. The first subcategory is characterized by the presence of ACPA autoantibodies, which could not be detected by already developed assays that, despite their high clinical sensitivity, are not able to detect all types of ACPA autoantibodies (43). Such patients might be ACPA positive and should belong to SP RA patients. Furthermore, patients falling into this sub-category exhibit a genetic background and exposure to environmental factors as in the case of SP RA patients (44). The second subcategory of RA patients has several circulating antibodies to unmodified native proteins/peptides (i.e. anti-RA33, anti-PTX3 and anti-DUSP11 antibodies) (45, 46). This sub-category of RA patients is clinically closely related to the SN RA patients. The third sub-category of RA patients are truly SN RA patients. Assay formats developed in a way to target a wide range of autoantibodies are essential to achieve enhanced clinical sensitivity and specificity. Finally, such diagnostic tests will allow for correctly stratifying RA patients into clinically relevant subcategories (30).

#### Anti-Carbamylated Protein Antibodies (anti-CarP)

RF- and ACPA-independent serum biomarkers are anti-CarP antibodies. They occur in approximately 25% to 50% of RA patients. Carbamylation is the reaction of isocyanic acid with the amino group of lysine, forming homocitrulline (Fig. 2). Isocyanates are formed from cyanates, thiocyanates and urea. This explains the increased carbamylation in patients with kidney diseases, in which urea accumulates in the body. Similarly, cyanate production by smoking and inflammation is the source of cyanates. Myeloperoxidase, the enzyme mainly stored in neutrophils, can convert thiocyanate to cyanate during inflammation.

As with RF, several subclasses have been identified, including all IgG subtypes but also IgA and IgM (47). Sensitivity and specificity for detecting RA are 30 to 50% and 90%, respectively (Table 2) (48). Anti-CarP can be detected in serum before the development of RA and have the potential to predict progression and joint erosion, especially in ACPA-negative RA. A combination of anti-CarP with ACPA and RF enhances the clinical specificity for RA from 65 to 100% to 98 to 100% (12).

A number of carbamylated proteins have been described, such as fibrinogen, vimentin, α-enolase, filaggrin, histones (i.e. very similar proteins as modified by citrullination) (38, 49, 50), some of which can identify up to 89% of cases of early RA (51). The reactivity profile of anti-CarP antibodies closely resembles that of ACPA. This overlap is attributed to the promiscuous binding properties of ACPA, since it was proved that many ACPA also recognize carbamylated antigens (30).

#### Anti-Acetylated Protein Antibodies (AAPA)

A third member of autoantibodies against modified proteins that is related to ACPA includes AAPA. These proteins are formed when acetyltransferases modify lysines present in proteins into acetylated lysines (Fig. 2). It is good to point to the fact that protein acetylation does not necessarily occur on folded proteins, since lysine acetylation was also described within intrinsically disordered regions in proteins (52). The same can also be true for other modifications of proteins.

AAPA are present in RA, and, like ACPA, can cross-react with various acetylated proteins and other modified proteins (i.e. homocitrullinated and citrullinated). AAPA reactivity is not present in ACPA-positive subjects. Thus, AAPA belong to a distinct category of autoantibodies against modified proteins (30). Typical targets modified by acetylation include filaggrin, vimentin, fibrinogen, α-tubulin, α-enolase and histones (38, 53).

#### Anti-PAD Antibodies

In summary, current biomarkers, objective clinical assessments and patients’ subjective experiences do not always align sufficiently. Consequently, there remains a pressing need to elucidate the precise pathogenic mechanisms underlying RA and to identify novel biomarkers that can improve diagnostics, prognosis and patient stratification.

Anti-PAD antibodies have been described in more than 35% of RA patients (54). There are five isoforms of anti-PAD antibodies providing different clinical information. For example, anti-PAD3 and anti-PAD4 can provide information about an increased severity and radiographic progression of the disease, while anti-PAD2 are associated with a mild RA form (12).

#### Anti-Mitochondrial Antibodies (AMA)

The reason why AMA were tested as RA biomarkers was that extracellular mitochondrial DNA has been associated with RA (12). AMA can predict severe RA disease for SP RA and SN RA patients. Antibodies against an outer mitochondrial protein were able to identify SN RA patients with severe RA type (55).

#### Advanced Glycation End-Products (AGE)

AGE together with malondialdehyde–acetaldehyde adduct (MAA)-modified proteins may under certain circumstances be targets for specific antibodies (56, 57). Analyses of 648 RA and 538 non-RA patients showed the presence of antibodies against AGE in 45% of RA patients and in 30% of control participants. These antibodies were also associated with radiological progression in serum-negative individuals (58).

#### Anti-RA33 Antibodies

Another potential area for biomarker discovery is related to the adverse effects of immune checkpoint inhibitors (ICIs). It has been observed that cancer patients treated with ICIs may develop an RA-like condition (59). Closer analysis has shown that almost all of these cancer patients are SN RA patients (60). Anti-RA33 antibodies (against heterogeneous nuclear ribonucleoprotein (hnRNP) A2/B1 involved in mRNA splicing) have been identified in the cancer patients treated with ICIs. It has been found that approximately 11% of people suffering from symptoms of RA induced by this treatment had anti-RA33 detected but not a single one who was treated without adverse effects of this kind (61). Anti-RA33 as an RA biomarker suffer from low sensitivity of 32 to 33%, despite a specificity of 90% (Table 2) (12, 62).

#### Long Pentraxin 3 (PTX3)

Long pentraxin 3 (PTX3) has also been studied as an RA biomarker. It is a protein whose concentration increases during inflammation and was significantly more expressed in the SP RA patients than in the SN RA patients. It is produced by several cells, including synovial fibroblasts in RA patients. The level of PTX3 was positively associated in synovial tissue with DAS28 and CRP (63). A combination of two biomarkers, i.e. anti-PTX3 with anti-DUSP11 moderately enhanced the diagnostic sensitivity to 38% (sensitivity for anti-PTX3 of 28% and for anti-DUSP11 of 32%) with specificity of 89% for RA patients, irrespective of the ACPA status (46). Meta-analysis based on 60 papers focused on an evaluation of the potential of pentraxin-3 as an RA biomarker (64). The study mostly showed an increase in the level of pentraxin-3 associated with RA (64).

#### Galectins

Galectins comprise a family of 15 small, soluble lectins characterized by their ability to specifically recognize and bind galactose-containing glycans (65).

Galectin 1 showed very promising discrimination power as a diagnostic biomarker in serum with an AUC of 0.95 (sens.: 85%; spec.: 98%) (66). Galectin 1 serological levels did not positively correlate with clinical disease parameters (66, 67, 68), but another study showed a positive correlation with ESR and DAS28 (69). Another study showed a significantly lower AUC of 0.76 (sens.: 71%; spec.: 79%) (67) or 0.82 (sens.: 80%; spec.: 73%) (69), when considering galectin 1 as an RA biomarker.

Galectin 3 also showed a promising discriminatory power with AUC = 0.88 (sens.: 85%; spec.: 71%) (69). A meta-analysis based on 12 published studies confirmed the potential of galectin 3 as a diagnostic and prognostic RA biomarker with the possibility to also become a therapeutic target (70). Galectin 3 levels in plasma and synovial fluid were linked with RA activity, and a decrease in galectin 3 levels predicted decreased long-term RA activity (71).

Galectin 4 was also considered a good RA biomarker with a sensitivity of 93% and a specificity of 79% (68).

The level of galectin 9 was elevated in human plasma of RA patients, when compared to HI, hence could be used as a diagnostic biomarker and correlated with RA disease activity (72).

The authors of the above-mentioned studies did not specify if galectins, when detected, were in any complex with glycoprotein-based binding partners.

#### Other Novel RA Biomarkers

The clinical performance of novel RA biomarkers presented in this section is summarized in Table 4.

Leucine-rich α-2-glycoprotein 1 (LRG1) is associated with inflammation and RA progression (73). The study involving human serum from 78 RA patients and 20 HI showed a higher level of LRG1 in RA patients with a discriminatory power represented by an AUC of 0.795. The level of LRG1 decreased 12 weeks after biologics treatment, and LRG1 positively correlated with BMI and CRP levels in RA patients. The authors concluded that LRG1 could be used for RA disease monitoring and to predict the outcome from using biologics (73). The other study showed a positive correlation of LRG1 with several clinical parameters such as clinical disease activity index (CDAI), CRP and ESR with a conclusion that LRG1 could be used for an evaluation of RA disease activity (74).

Osteopontin (OPN), a glycoprotein associated with inflammation and immune regulation, is a promising RA biomarker (75). The clinical potential of OPN was investigated in a meta-analysis involving 37 studies. The results indicate that OPN is elevated in several rheumatic diseases, with a moderate increase in OPN in RA. There is, however, quite a high variability of the OPN level in RA patients, calling for further studies to evaluate the contribution from other factors, including disease severity, comorbidities and treatment effects (75).

The clinical potential of fetuin A, a glycoprotein produced by the liver, was investigated in a meta-analysis including 19 studies (76). The review paper showed a decrease in fetuin A levels associated with RA (76). Fetuin A levels were negatively correlated with ESR and DAS28-ESR (i.e. DAS28 combined with ESR) and also showed some diagnostic potential in the other study (77).

Anti-citrullinated scavenger receptor A peptide antibody (anti-CSP) was validated in a multicenter study to prove its potential as an RA biomarker (78). The results showed its promising clinical performance with sensitivity of 59% and specificity of 94% (AUC = 0.78–0.88); a combination of anti-CSP with ACPA showed increasing discriminatory power with sensitivity of 85% and specificity of 92% (AUC = 0.85–0.97). Furthermore, the study revealed a distinct glycosylation of anti-CSP with its pro-inflammatory role and pathogenic effect in RA. Anti-CSP antibodies showed promise for the diagnosis of, especially, SN RA patients and early-stage RA disease (78).

The upper zone of the growth-plate and cartilage matrix-associated protein (UCMA) is a member of the vitamin K-dependent protein family, showed the ability to discriminate between RA patients and HI with an AUC of 0.76 (79). Other proteins investigated in the study were compared to UCMA as an RA biomarker with the following discriminatory power represented by AUC values: COMP (cartilage oligomeric matrix protein; AUC = 0.75), TNFα (AUC = 0.89) and IL-6 (AUC = 0.63). UCMA together with COMP and TNFα have the potential to be used as a diagnostic or predictive RA biomarker in the management of RA patients (79).

Glycoprotein tenascin C exhibited a promising discriminatory power to distinguish RA from HI with an AUC of 0.98 (sens.: 97%; spec.: 100%) and for discrimination of SN RA *versus* HI with an AUC of 0.97 (sens.: 93%; spec.: 100%) (80). A combination of tenascin C with RF and ACPA could be used to distinguish SN RA from SP RA (80).

Metalloproteinase 3 was tested as an RA biomarker for discrimination of SN RA *versus* HI by analysis of serum samples from 145 SP RA, 145 SN RA and 38 HI (81). The results indicate that metalloproteinase 3 can be applied to distinguish SN RA patients from HI with an AUC of 0.93 (sens.: 84%; spec.: 92%). Furthermore, metalloproteinase 3 can also act as a biomarker to distinguish RA patients from HI with an AUC of 0.92 (sens.: 84%; spec.: 92%) and to distinguish SP RA from HI with an AUC of 0.92 (sens.: 85%; spec.: 92%). Furthermore, the biomarker can be applied to RA disease monitoring, for RA prognosis and as a potential target for therapeutics (81).

#### Exosomal Proteins

Exosomes belong to extracellular lipid bilayer-based vesicles, produced by various cell types into the extracellular space. Exosomes play critical roles in intercellular communication and are involved in numerous physiological and pathological processes. Moreover, exosomes are considered to be a source of precious cargo, i.e. biomolecules, which can be promising biomarkers (82, 83). In this section, we do not cover exosomal miRNAs as RA biomarkers; the reader is advised to read the recent review paper on this topic (4).

It has been proved that two proteins present in the exosomes, that is, amyloid A and lymphatic vessel endothelial hyaluronic acid receptor-1 scan be applied as biomarkers for monitoring RA disease activity (84).

A high-resolution quantitative proteomics was able to reliably quantify 682 exosomal proteins, of which 26 proteins were upregulated and 31 downregulated in RA patients (85). Serum amyloid A1 protein and alarmin S100A9 were strongly associated with RA, and serum amyloid A1 and serum amyloid A2 proteins correlated with disease activity (DAS28, CRP and ESR). The study clearly showed that fewer proteins (343) were reliably quantified in blood serum than exosomes, and only 42% of exosomal RA proteins were quantified in blood serum (85). Hence, exosomes could be a reliable source of precious RA biomarkers with an enriched concentration in comparison with blood serum.

It was also found that exosomal surfaces exhibited a higher expression of citrullinated antigens in RA patients than in HI (86). Three proteins, i.e. vimentin, glycolytic enzyme α-enolase 1 and collagen type II were identified as citrulline carriers and such antigens correlated positively with disease activity (86). Two other studies revealed differences in the proteomic prolife of exosomes from RA patients, when compared to HI, with their potential as RA biomarkers (83, 87).

The RA patients had higher levels of syndecan-1 than HI, with the protein level evaluated as an RA biomarker with sensitivity of 84% and specificity of 52% (88).

#### Multi-Biomarker-Based Assays

Initially, 130 candidate biomarkers were selected from extensive literature screens, bioinformatics databases, mRNA expression, and protein microarray data, of which 25 were subsequently selected for algorithm training. Finally, the multi-biomarker disease activity (MBDA) test used 12 protein biomarkers to generate the disease score (89). The assay was run in ELISA format and afforded an AUC of 0.89 for discrimination between patients with low and those with moderate/high RA activity. Furthermore, the MBDA score exhibited a positive correlation with the DAS28-CRP score (89). The MBDA score also showed a positive correlation with DAS28-ESR, SDAI (simplified disease activity index) and CDAI scores; the MBDA score reflects current clinical disease activity and enables longitudinal monitoring of changes over time (90). A review of the literature and meta-analysis of data generated with the MBDA test concluded that the test was of high value in the management of RA patients, especially for monitoring disease activity and predicting radiological progression (91, 92). The good clinical performance of the MBDA test was a trigger in establishing the company Crescendo Bioscience in 2002 in order to start commercialization activities with regard to the product. Crescendo Bioscience was later (2014) acquired by Myriad Genetics, and the analysis is offered as a service in their own certified labs.

#### Computational Methods and Artificial Intelligence (AI)

The autoimmune response in RA patients is very complex and not that well understood (30). This is why multi-omics data (genomics, transcriptomics, proteomics, metabolomics, epigenomics, and microbiome) integrated with artificial intelligence (AI) has the potential to enable better RA diagnostics, patient management/stratification and therapy selection. However, successful translation from basic research to clinical practice requires proper analytical and clinical validation of the biomarkers, regulatory evidence, explicability, analysis of cost-effectiveness and integration with clinicians and electronic health record (EHR) systems (28).

Comprehensive reviews published recently offer an in-depth analysis of the state of the art regarding the application and benefits of AI and machine-learning algorithms in RA (93, 94, 95, 96, 97, 98). Such approaches can help identify relationships between different types of data that were previously unrecognized and can be used for RA diagnostics, disease monitoring, evaluation of risk of complications, drug discovery, therapeutic response, and so on.

For example, a deep learning model was used for prognosis of the status of the disease in RA patients at their next clinical visit (99). Structured data from EHRs, medications given, patient demographics, lab results and prior disease activity score were taken into account. When the model was run at one hospital, an AUC of 0.91 was reached, and when it was run at another hospital, an AUC of 0.74 was determined. The findings indicate that it is feasible to develop accurate AI models for predicting complex diseases such as RA and that these models can also be successfully applied in different hospitals and for different patient populations (99).

With the development of computing capacity as well as the gradual application of AI to practical life, new approaches are opening up that facilitate new connections between already known entities and help identify new prospective biomarkers. For example, the artificial neural network helped reduce the number of inflammatory proteins to discriminate between RA and HI from 38 to 12 (100). Clinical parameters of discrimination varied depending on the number of biomarkers (2–12) used in the evaluation, with sensitivity of 80% to 93% and specificity of 92 to 100% (100).

Advanced analysis of LC-MS/MS spectra revealed 24 key proteins as potential RA biomarkers. The Random Forest machine-learning model helped identify the most significant potential RA biomarkers and reduced their number to four. These proteins exhibited an AUC in the range of 0.78 to 0.84 (101).

A machine-learning algorithm was used for the selection of RA biomarkers from the pool of 1307 proteins to find a minimal set of proteins for disease discrimination (87). Eventually, 23 proteins were identified as contributing to early-stage RA diagnostics, affording an AUC of 0.93. The panel of biomarkers can be used for identification of pre-RA patients years before clinically detectable symptoms develop (87).

An artificial neural network was used for the mining of data generated by analysis of 10 different conventional RA biomarkers and analysis of glycans on antibodies in 10 different assay formats to evaluate promising biomarkers (102). A discrimination between SP RA and HI revealed that a combination of standard and glycan biomarkers was beneficial for the discrimination, while for discriminating between SN RA and HI, only glycan biomarkers proved to be promising (102).

Attenuated total reflectance Fourier transform infrared spectroscopy was used for the analysis of blood serum samples from RA patients and HI (103). Data obtained in the form of spectra were processed using machine-learning models combining partial least squares discriminant analysis and a support vector machine. The results indicate that RA patients could be distinguished from HI with an AUC of 0.87 in the validation set and an AUC of 0.67 in the test set (103).

**Fig. 3:**
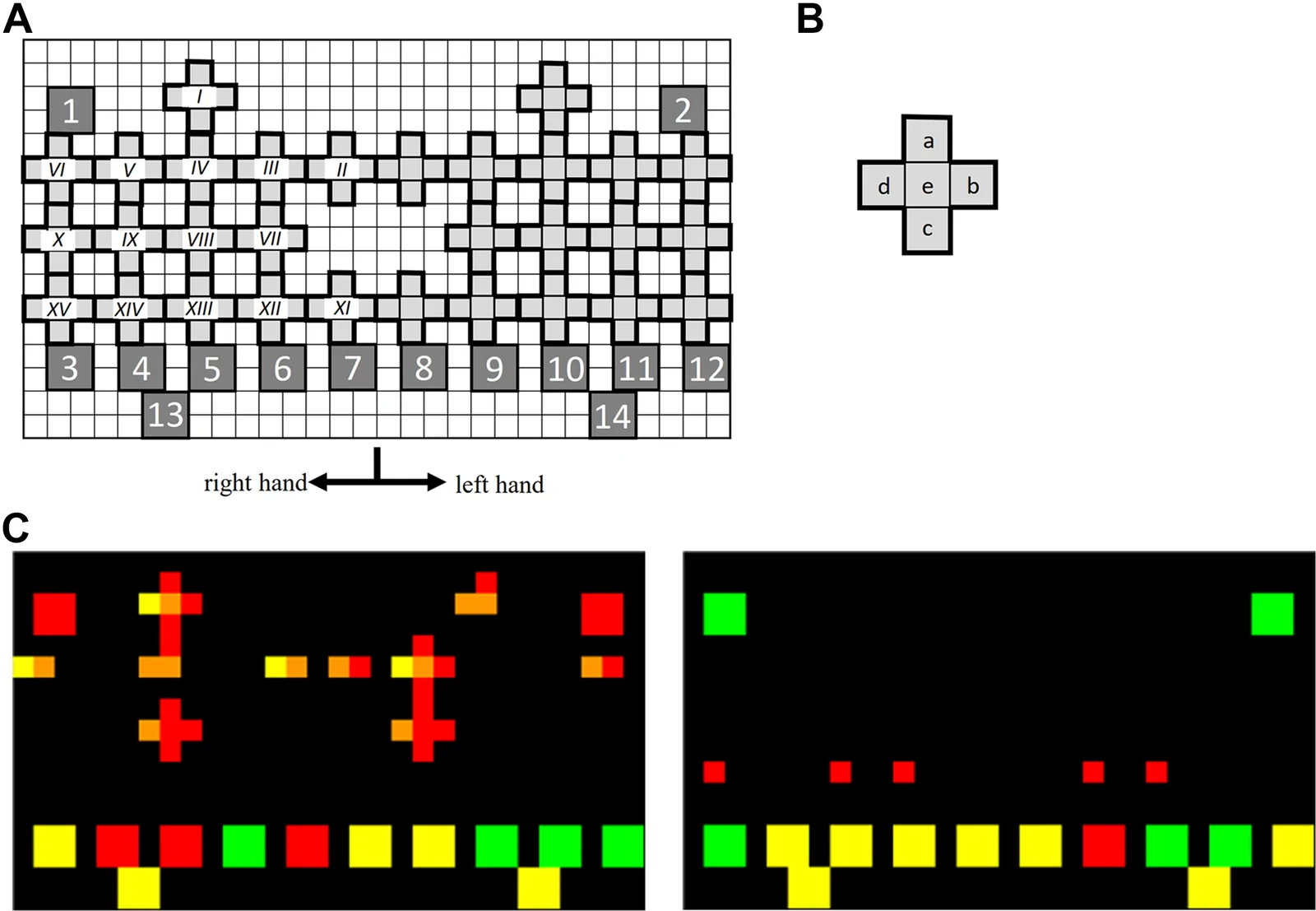
**Design of the 2D array image.***A*, the blocks in the 2D array show results obtained for each joint (I: wrist, II: MCP1, III: MCP2, IV: MCP3, V: MCP4, VI: MCP5, VII: PIP2, VIII: PIP3, IX: PIP4, X: PIP5, XI: IP, XII: DIP2, XIII: DIP3, XIV: DIP4, XV: DIP5). The 2D array is symmetrical with square blocks with number inside (1–14) expressing clinical information. *B*, The 2D array pattern consists of five squares labelled as a, b, c, d, and e, with each indicating joint information. *C*, representative images of RA (C-1) and of HI (C-2) are shown. RA = rheumatoid arthritis, MCP = metacarpophalangeal, DIP = distal interphalangeal, PIP = proximal interphalangeal, IP = interphalangeal. The image was taken from the open access publication (104).

A very interesting method for evaluating an array of clinical information was proposed by Fukae *et al.* (104). The authors transformed clinical data (i.e., the level of serological biomarkers and ultrasound of hands) to four colors based on a semi-quantitative evaluation. For example, RF level (IU/ml) was represented by colors as follows: green <15, 15 to 30 yellow, 30 ≤ red, and ultrasound grey scale score of both hands was represented by colours as follows: 1 = yellow, 2 = orange, 3 = red. Based on this, 2D array images were generated for both hands for every patient (Fig. 3). The convolutional neural network was then used to evaluate 1037 images (252 RA and 785 HI) as the training set. Finally, 50 images were evaluated to test the evaluation algorithm and compared with the classification provided by three rheumatologists. The model correctly classified 94, 90, 96, 98 and 98% of the individuals involved in the testing sets (10 RA + 40 HI) (104).

## Glycosylation

Protein glycosylation is a co- and/or post-translational modification in which complex oligosaccharides (glycans) are covalently conjugated to proteins. The glycosylation of proteins generates a substantial structural heterogeneity in the glycans attached to a given protein. Glycan structures encode biomolecular information reflective of the cellular state, with glycoproteins from distinct cell types and tissues exhibiting characteristic glycosylation patterns (105). Extensive research over several decades has further demonstrated that glycan compositions and structures are altered between normal physiological conditions and disease states. Consequently, glycoproteins and their associated glycans represent a valuable source of molecular information for disease diagnosis and clinical monitoring (106).

### Glycans

Glycans are complex carbohydrates attached to proteins and lipids that regulate numerous cellular processes (75, 107, 108, 109). Glycans attached to proteins can be classified into several categories based on the nature of the linkage between the glycan and the protein. These include *N*-glycans, which are attached to the amide nitrogen of asparagine residues; *O*-glycans, which are linked to the hydroxyl groups of serine, threonine, or other hydroxylated amino acids; and less abundant forms such as *C*-glycans, which are covalently attached via a carbon-carbon bond to tryptophan residues, and the relatively rare *S*-glycans, which are linked through a carbon–sulfur bond to cysteine residues (110). In addition, glycans may exhibit varying degrees of branching, forming bi-antennary, tri-antennary, or more highly complex antennary structures (110). Recent studies indicate that RNA can also be modified by *N*- and *O*-glycans (111, 112, 113, 114).

**Fig. 4:**
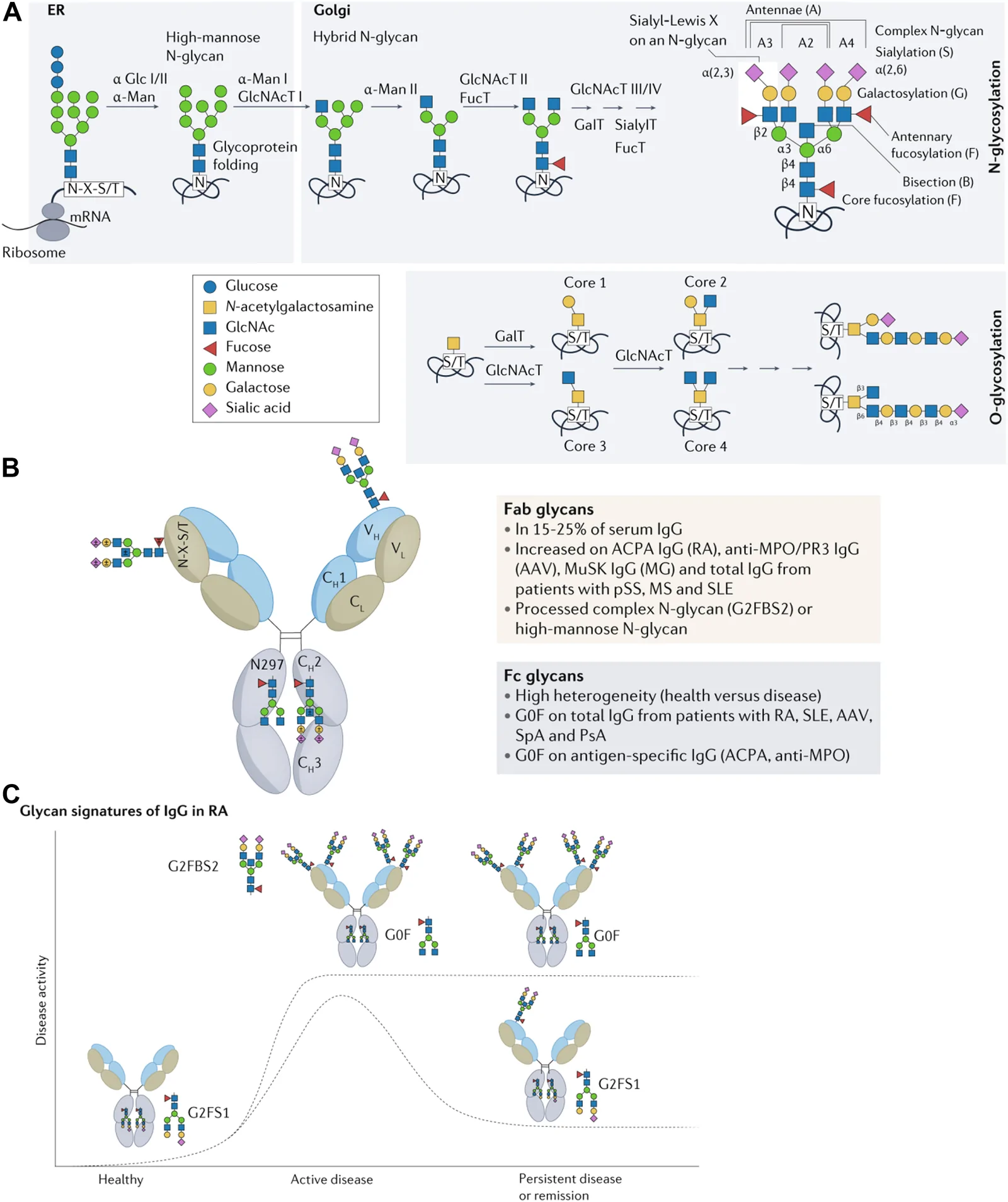
**Biosynthesis of protein glycans and the IgG-specific glycan signatures in rheumatoid arthritis.***A*, *N*-linked glycans are linked to protein via –NH_2_ group. Formation of a precursor of *N*-glycan is done in the endoplasmic reticulum (ER). Initially, removal of terminal glucose/mannose residues by the enzymes is followed by addition of *N*-acetylglucosamine (GlcNAc) by the action of the other enzyme (GlcNAcT). Then, from the protein mannose units are trimmed in the Golgi apparatus with formation of complex-type bi-antennary (A2), tri-antennary (A3) and tetra-antennary (A4) *N*-glycan structures. *O*-linked glycans are attached to proteins via –OH group. *B*, schematic representation of an IgG molecule. The fragment crystallizable (Fc) domain is 100% *N*-glycosylated at N297, and the fragment antigen-binding (Fab) domain is 15 to 25% *N*-glycosylated at N-X-S/T consensus motifs. Fab glycosylation is increased on anti-citrullinated protein antibodies (ACPA) IgG from patients with RA. Rheumatic-disease-specific IgG Fc glycans are characterised by the presence of fucose but not galactose (G0F). *C*, the glycan signatures of ACPA IgG in RA. Fc galactosylation decreases and processed Fab glycosylation (represented by ‘G2FBS2’, where G is galactose, F is fucose, B is bisecting GlcNAc and S is sialic acid) increases towards disease onset and is associated with disease severity. FucT, fucosyltransferase; GalT, galactosyltransferase; PsA, psoriatic arthritis; SialylT, sialyltransferase; SpA, spondyloarthritis. Reproduced with permission from Springer Nature from Ref. (106).

Glycan biosynthesis is mediated by writers (glycosyltransferases) and erasers (glycosidases), while glycan recognition is carried out by readers (lectins/agglutinins) (107, 115). An initial phase of synthesis of precursors for *N*-glycans is carried out in the endoplasmic reticulum (ER), and a final processing/maturation of *N*-glycans is completed in the Golgi apparatus (Fig. 4*A*). Synthesis of *O*-glycans proceeds in the Golgi apparatus (Fig. 4*A*). The surface of every human cell is heavily coated with glycans, with an estimation that each glycoprotein exists, on average, in approximately 50 different glycoforms (115). Due to their fundamental role in cellular function, few biological processes can be fully understood without considering glycans. As has been stated, “a protein without its glycans is like a plate without food; structurally defined but functionally incomplete” (115, 116).

### Analysis of Glycans

Analysis of glycans remains challenging and can be performed using three distinct approaches: 1. retention time-based assay formats, i.e. liquid chromatography; 2. charge- or mass-based analysis, i.e. capillary chromatography or mass spectrometry; 3. affinity-based glycan determination using either lectins or anti-glycan antibodies (117). Approaches 1 and 2 are often combined to make analysis of glycans even more robust and to obtain complementary information.

While approaches 1 and 2 can provide exact structural glycan analysis, such methods are rather complex, requiring costly infrastructure, labor- and time-intensive protocols, and sometimes the data evaluation is not trivial and requires experienced operators. However, strategies to make instrument-based glycan analysis simpler and more robust are constantly evolving (105, 118, 119, 120, 121, 122, 123, 124).

On the one hand, affinity-based approaches offer significant advantages over instrument-based glycan analysis formats, that is, simple sample preparation, high or moderate assay throughput, and high analytical sensitivity (i.e., sub-nanogram detection levels). On the other hand, affinity-based approaches are limited by the repertoire of lectins and anti-glycan antibodies and cannot provide quantitative information and provide only limited information about the structures of glycans detected. Accordingly, there are studies combining instrument-based and affinity-based methods for glycan analysis, especially when searching for novel glycan-based biomarkers (125).

### Glycosylation in RA

Encouragingly, we are entering an era in which glycosylation is increasingly integrated into the biological context. A recent study shows that we have computational tools to predict *N*-glycan abundance from glycogene expression (i.e., genes associated with sugar transporters, nucleotide sugar synthesis, dolichol pathway, glycosyltransferases, glycosidases and lysosomal degradation) to better understand the cellular machinery behind glycosylation and design novel diagnostic and therapeutic protocols (126).

Alterations in glycosylation are already described as a hallmark of cancer. Even further, another perspective suggests that glycans contribute to every recognized hallmark of cancer (115, 127). The defined glycosylation change in IgG as a sign of RA was already characterized in 1985 (128). The study identified a significantly lower level of galactosylation in RA patients by the analysis of 1400 glycans released from IgGs of 46 serum samples (128). The first study showing Fab glycosylation and its relevance to RA was published in 1996 (129). Recently, the Fab glycosylation of ACPA IgG autoantibodies and its level have been identified as an early hallmark of RA development (15, 130). Another study of 10,000 individuals showed a direct link between plasma *N*-glycome and anti-inflammatory proteins (131). Accordingly, we may conclude that changed glycosylation is also a hallmark of RA.

It is estimated that at least half of the proteins encoded in the human genome are glycosylated. A comparison of total serum *N*-glycans between healthy subjects and those with RA revealed several differences. These include an increase in fucosylation of tri- and tetra-antennary glycans together with the presence of α2,3 sialic acid (106), indicating the presence of sialyl-Lewis X containing motifs within branched *N*-glycans. Overexpression of this Lewis motif has been described in several inflammatory processes for proteins including α1-acid glycoprotein, haptoglobin, α1-antichymotrypsin and transferrin (132). Another feature is the positive correlation between RA activity and the amount of sialylated and fucosylated tri-antennary glycans. Conversely, the activity is negatively correlated with galactosylated bi-antennary (A2) *N*-glycans (106).

**Fig. 5:**
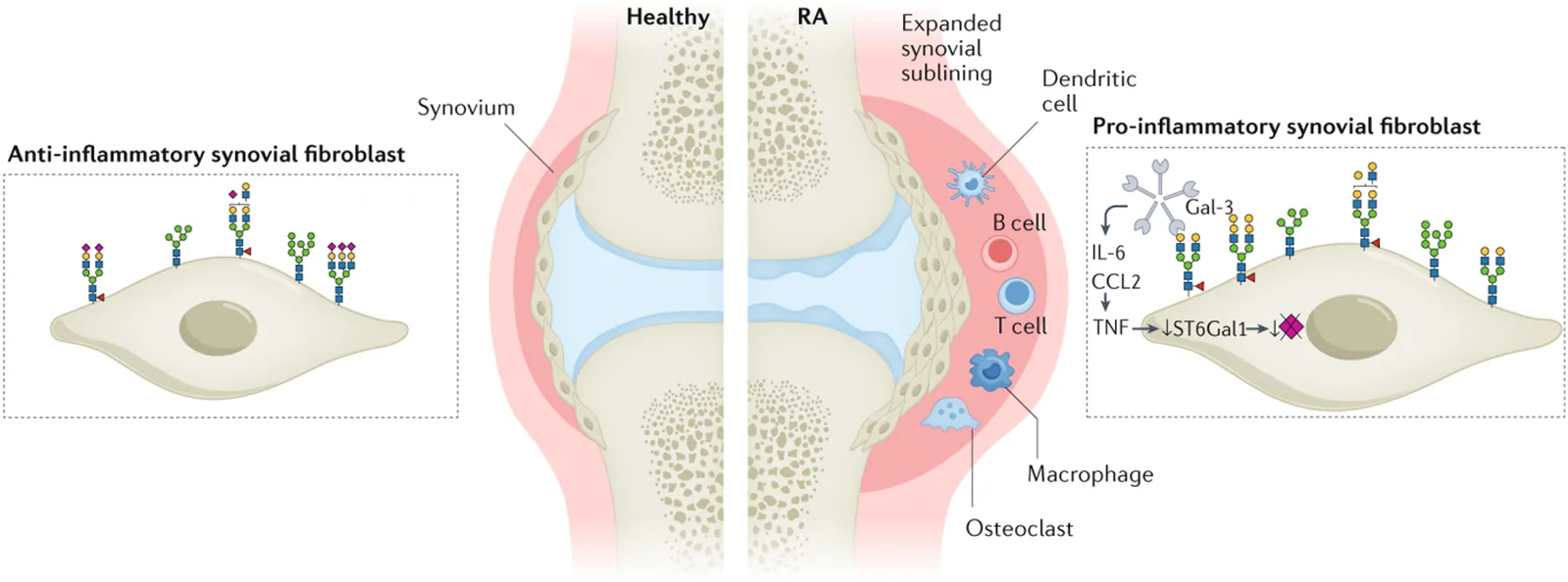
**The synovial fibroblast contains especially *N*-glycans composed of high-mannose units and short bi-antennary *N*-glycans, showing differences in terminal sialylation for both RA patients and HI.** In RA, TNF (tumour necrosis factor α) stimulates downregulation of ST6Gal1 (β-galactoside α-2,6-sialyltransferase 1), what result in decreased levels of sialic acid with subsequent switch to a more pro-inflammatory condition. *N*-glycans present on the surface of synovial fibroblast may interact with galectin-3 (Gal-3), inducing production of pro-inflammatory cytokines (IL-6, CCL2), which in turn upregulate the TNF expression and thus desialylation of the synovial fibroblast surface (in a vicious cycle). Reproduced with permission from Springer Nature from Ref. (106).

One of the best-described changes in glycosylation associated with RA is the decreased galactosylation of *N*-glycan present in the Fc domain of IgGs. IgGs with *N*-glycans without any galactose are pro-inflammatory molecules, since such IgGs increasingly interact with Fcγ receptors and activate the complement system, with a final activation of the immune system (133). Changed glycosylation is associated not only with IgGs, but also with synovial fibroblasts and T cells (Fig. 5) (133). Recently, an antibody recognizing agalactosylated IgGs was developed and used in the diagnostics of chronic inflammatory disease but was not tested for RA patients (134).

### Glycosylation of Immunoglobulins

Immunoglobulins are made up of different chains, that is, two heavy chains and two light chains connected by disulfide bonds. The Fab fragment, that is, antigen-binding fragment, binds to different antigens. The Fc fragment of immunoglobulins binds to specific ligands such as cellular receptors and the C1 component of the complement, responsible for various effector functions (135).

IgG Fc *N*-glycosylation remains highly stable in frozen serum/plasma samples (136). Short exposure to ambient temperature, visible light, or muliple freeze-thaw cycles does not significantly affect the glycosylation patterns. These findings suggest that sample storage requirements for IgG glycosylation analysis are less stringent than those typically necessary for other omics studies (136). Another study confirmed a high stability of glycosylation of IgG in human serum and plasma, i.e. stability at ambient temperature for up to 2 weeks, confirming the attractiveness of glycosylation of IgG as a clinical RA biomarker (136).

#### *N*-Glycosylation of IgGs

IgGs produced by B cells are the most dominant types of immunoglobulins (∼75%) with the highest level among all proteins in blood (∼10 mg/ml) in human serum. There are 4 types of IgGs with the following abundance: IgG1 (60%), IgG2 (25%), IgG3 (10%), and IgG4 (5%) (137). The number of disulfide bonds in the hinge region differs among IgG isoforms: IgG1 and IgG4 each contain two interchain disulfide bonds, IgG2 contains four, and IgG3 contains eleven (137).

**Fig. 6:**
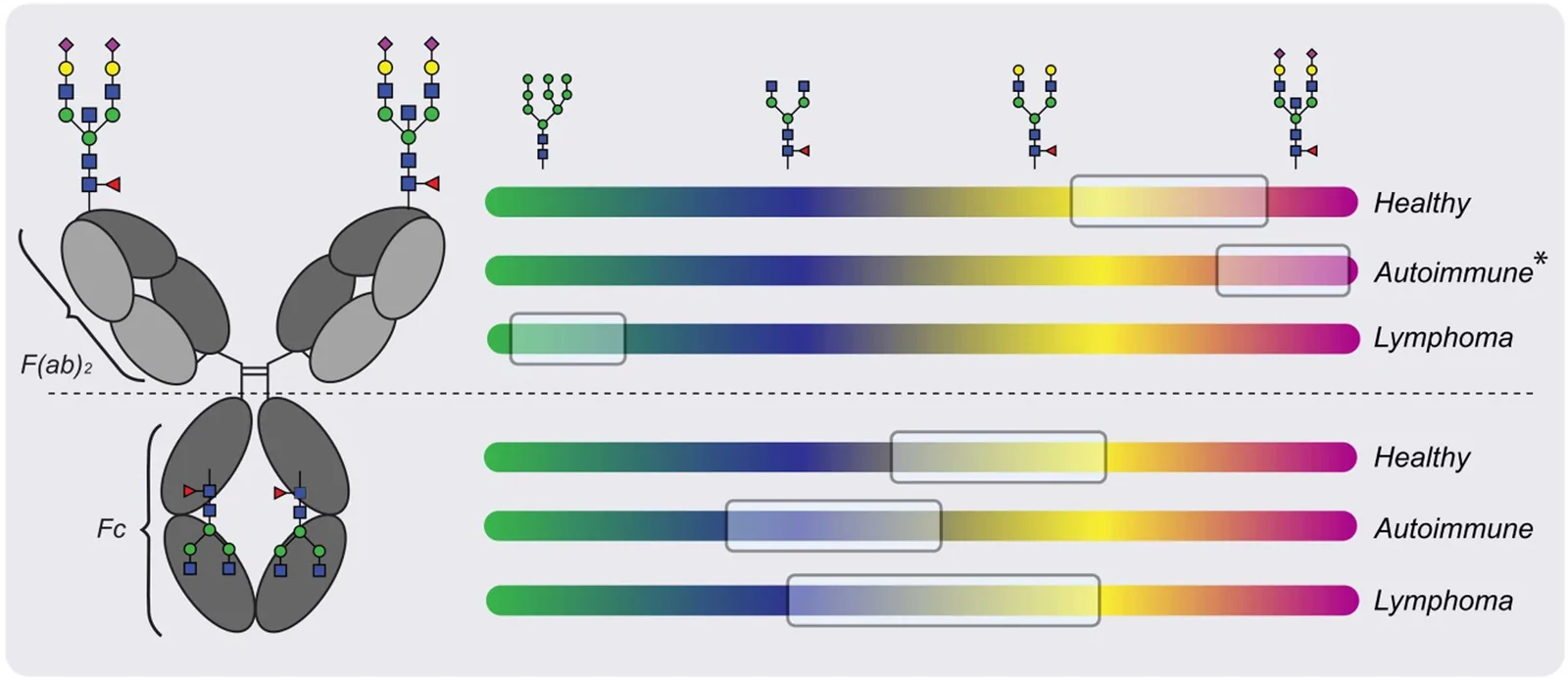
**Glycosylation is different when comparing Fab and Fc domains.** Colored bars show types of glycans present in various diseases: high-mannose glycans are in *green*, agalactosylated glycans in *blue*, fully galactosylated glycans in *yellow*, and sialylated glycans in *magenta*. Fc: fragment crystallizable; F(ab)_2_: fragment antigen-binding dimer. The image was taken from the open access publication (139).

There are 3 different sites occupied by glycans within IgGs, that is, *N*-glycans in the Fc domain of IgGs (Fig. 4*B*), *N*-glycans in the Fab domain of IgGs (Fig. 4*B*); and *O*-glycans in the hinge domain of only IgG3. There are significant differences in the composition of *N*-glycans when the Fc domain is compared with the Fab domain (see Table 5 and Fig. 6) (135, 138, 139).

**Table 5:** Composition of *N*-glycans Present Either in the Fc and the Fab Fragment of IgGs (Data Taken From Refs. (135, 138))

Glycans	Content in the Fc fragment (%)	Content in the Fab fragment (%)
Di-sialylated	1	52
Bisecting	10–15	45
Core fucose	90	70
Di-galactosylated	15–40	94
High mannose	<1	4

##### N-Glycosylation in the Fc Domain of IgGs

All IgGs circulating in the bloodstream are N297-glycosylated in the CH2 domain fragment crystallisable (Fc) domain (140). The glycans present in the Fc fragment of IgGs are hidden within the cavity between heavy chains and stabilise the conformation of the Fc fragment of IgG. The *N*-glycans present in the Fc domain are predominantly complex bi-antennary type glycans which are (106): a) commonly core-fucosylated, b) with varying antennal galactose content (G0, G1 and G2 for none, one or two residues), c) to a lesser extent bisecting GlcNAc (GlcNAc attached to the mannose residues), d) or terminally-linked sialic acid. High-mannose and hybrid *N*-glycans can also be present on the Fc fragment (<1% of IgGs) (135).

In total, 36 different glycan structures present in the Fc fragment were described (135). Most of the IgGs in the Fc fragment contain GlcNAc in both arms, 90% contain core fucose, 40% of *N*-glycans contain only one galactose molecule, and only 1% of *N*-glycans contain two sialic acid residues (Table 5) (135).

**Fig. 7:**
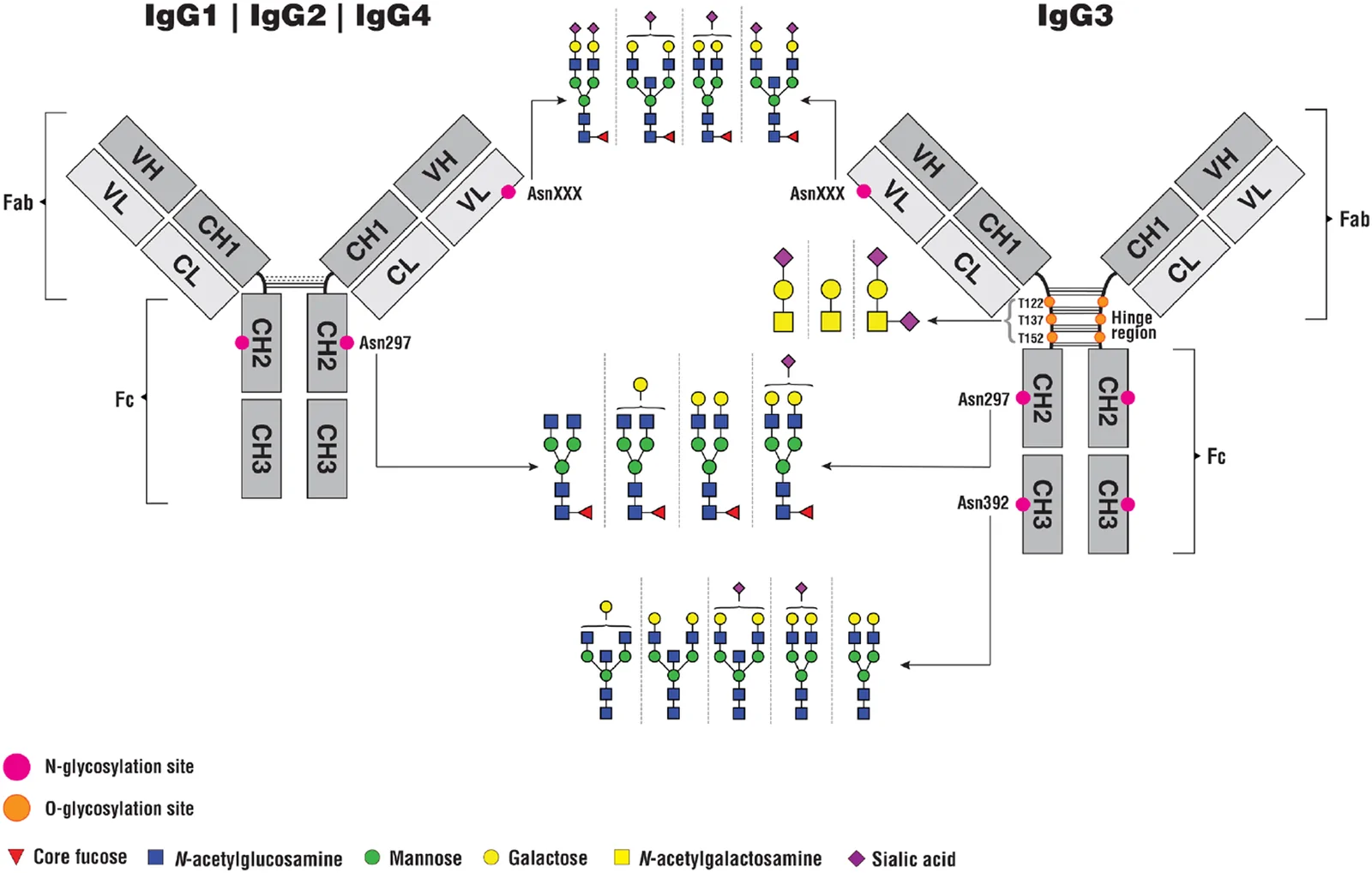
**Sites of glycosylation on IgG1-4 subclasses and the structure of glycans present at these sites.** The most abundant glycan types identified at each glycosylation site are shown here. The image was taken from the open access publication (138).

Three out of four subclasses of IgG, that is, IgG1, IgG2, and IgG4, are very similar to each other in terms of glycosylation with the Asn297 *N*-glycosylation site present on the Fc domain of the IgG and the other *N*-glycosylation site present in the Fab domain (Figs. 4*B* and 7) of IgG.

The IgG3 subclass is different from those three subclasses since IgG3 contains an additional *N*-glycosylation site in the Fc domain, i.e. Asn392 and, most importantly, *O*-glycosylation sites in the hinge region of IgG3 (Fig. 7) (138). The *N*-glycosylation site at Asn297 can be occupied by 30 different glycan structures significantly affecting the function of IgGs. Furthermore, structural complexity at Asn297 can be enhanced since the two *N*-glycans present within the Fc domain of IgGs do not need to be identical (138). The *N*-glycans present at Asn297 are mainly complex glycans with a minority of glycans being of high-mannose or hybrid type of *N*-glycans (only 1% of total *N*-glycans) (138).

While the site Asn297 is practically always occupied by *N*-glycans, the site Asn392 in IgG3 contains *N*-glycans only in 12% of all IgG3s (138). *N*-glycans at Asn392, when compared with the *N*-glycans at Asn297, lack core fucose and predominantly contain bisecting GlcNAc (138). The role of the *N*-glycans occupied at Asn392 in IgG3s is even less understood than the role of *O*-glycans in IgG3s (138).

Current knowledge suggests the following facts regarding the association of changed glycosylation within the Fc domain of IgGs and RA (15, 106, 141, 142): a) the level of galactosylation, and especially the level of agalactosylated, is associated with RA development, progression, disease activity and relapse of RA; b) a transient increase in the content of galactosylated residues during pregnancy with a decrease in galactosylation after delivery; c) the level of sialic acid in RA patients is lower with a decrease in sialylation observed prior to the onset of the RA disease; d) no correlation has been found between core-fucosylation and RA disease activity or inflammation.

##### N-Glycosylation in the Fab Domain of IgGs

An unexpected feature that ACPA carry over 90% of *N*-linked glycans in the Fab domain was identified in 2016 (143). Glycans can be present in the Fab domain of ACPA due to formation of the site following somatic hypermutation in ACPA-producing B cells. Glycans in the Fab domain of ACPA mainly consist of complex-type carbohydrates having terminal sialic acids (30). Interestingly, Fab glycans are associated with anti-inflammatory effects of intravenous immunoglobulin treatment therapy (138).

Unlike the Fc domain of IgGs, the Fab fragment contains a more diverse set of *N*-glycans (Table 5). This is due to the better accessibility of the glycan site on the Fab fragment for the action of glycan-processing enzymes, i.e. glycosyltransferases and glycosidases, than the glycan site on the Fc fragment (135). Fab glycosylation of IgGs is dependent on the subtypes with the following values: IgG1 (11–18%), IgG2 (10–16%), IgG3 (11–16%) and IgG4 (40–50%) (144). An increased glycosylation of the Fab fragment of IgGs was observed in autoimmune diseases including RA disease in comparison with healthy controls (135).

The glycans present in the Fab domain of IgG antibodies affect a wide range of IgG functions, including stability, solubility, half-life, antigen-binding, formation of immune complexes and immune response (135, 138, 139). The study investigating the impact of Fab-associated glycans on ACPA has shown that the presence of glycans in the Fab domain is linked to a reduced binding affinity to antigens (145). Enzymatic release of sialic acid residues from Fab glycans enhances antigen-binding, whereas a complete removal of the Fab glycan results in a more pronounced increase in binding affinity (145). By contrast, for at least one reported ACPA, the removal of Fab glycans was associated with decreased antigen binding, indicating antibody-specific effects (145).

The presence of glycans on ACPA positively correlates with RA disease progression and therapeutic outcomes for RA patients (139). The presence of glycans in the Fab fragment of ACPA significantly increases the risk of developing RA later in life (i.e. 10 years later) with an increasing level of Fab glycans towards the RA disease onset, and then it remains stable when the RA disease is present (Fig. 4*C*) (30, 139).

When the level of Fab glycans is fairly low at the time of RA onset, RA patients have an increased chance to achieve remission without any treatment (139). Furthermore, the presence of *N*-glycans in the Fab domain of ACPA in ACPA-positive individuals without RA is linked to the progression towards RA (30). Fab glycosylation of IgGs might have predictive potential since lower Fab glycosylation at the time of RA onset is associated with a greater probability of achieving sustained remission without the need for drug treatment (15).

Fab *N*-glycosylation of ACPA contains a greater amount of di-galactosylated (84% *versus* 73%) and disialylated (44% *versus* 27%) *N*-glycans than the Fab *N*- glycosylation of total IgG in RA patients (146). It is reported that the Fab glycosylation of IgG (IgG1 and IgG4) antibodies was especially elevated in patients with a chronic form of RA disease compared with the acute form of RA disease (147).

The intravenous injection of mRNA encoding two enzymes, i.e. galactosyltransferase and sialyltransferase, was successfully used in shifting the immune response in rats from a pro-inflammatory to anti-inflammatory state, similar to intravenous immunoglobulin (IVIG) therapies (148). In contrast to the total plasma IgG1 antibody repertoire, the ACPA IgG1 sub-repertoire is characterized by an enrichment of antibodies bearing one, two or multiple Fab glycans, with distinct glycovariants detectable within the same clonal lineage. These data indicate that the autoantibody response in RA is highly complex, patient-specific, and dominated by a relatively limited number of antibody clones (149).

Modification in Fab glycosylation of ACPA IgG autoantibodies may serve as a biomarker for the preclinical phase of the disease and holds a potential for guiding early, targeted interventions aimed at preventing the onset of RA (15). On the other hand, one study suggests that a subset of ACPA is associated with protection from RA with therapeutic potential (150).

It is estimated that 50% of anti-CarP antibodies IgGs are glycosylated in the Fab fragment and 60 to 90% of ACPA are glycosylated in the Fab fragment, while for HI 10 to 25% of total IgGs are glycosylated in the Fab fragment (139). An increased glycosylation in the Fab region is also observed for other autoantibodies, such as those against myeloperoxidase (151).

##### O-Glycosylation of IgGs

The occupancy of *O*-glycans on all three glycosylation sites within IgG3 is around 10% and preferentially occupied by core 1 *O*-glycans (138). Typical *O*-glycans contain non-, mono- and di-sialylated core 1 type *O*-glycans (Fig. 7) (138, 152). IgG can be *O*-glycosylated in the hinge region of the IgG3 subclass, since the hinge region of IgG3 is elongated, representing ∼10% of total IgGs (152, 153, 154). The function of *O*-glycans present in the hinge domain of IgG3 remains obscure. There are suggestions that it can protect the hinge domain from being cleaved by proteases and stabilise the extended conformation of the hinge domain, but it can be involved in the development and progression of some diseases (138).

#### IgA Glycosylation

IgA is known as the most heavily glycosylated immunoglobulin. However, in contrast with the well-established role of IgG glycosylation, the pathogenic mechanisms associated with IgA glycosylation in related diseases remain unclear (155). IgA is present as two different isotypes, i.e. IgA1 (84%) and IgA2 (16%). Both isotypes contain multiple *N*-glycosylation sites, with IgA1 having an additional 9 *O*-glycosylation sites in the hinge region due to a longer hinge domain than IgA2 (155). Aberrant *O*-glycosylation of IgA is predominantly associated with kidney diseases such as IgA nephropathy and IgA vasculitis. In patients with IgA nephropathy, galactose-deficient IgA1 are recognized by anti-glycan autoantibodies, triggering the formation of polymeric IgA1-containing immune complexes. These complexes subsequently deposit within the glomerular mesangium, leading to kidney inflammation and scarring (156).

Differences in the glycosylation of IgA1 and IgA2 have been linked to 3D structural differences of these two types of IgA, rather than by different expression of glycan-processing enzymes (157).

##### N-Glycosylation of IgA

IgA1 has only two *N*-glycosylation sites (N144 and N340), while IgA2 has 5 *N*-glycosylation sites (N47, N92, N131, N327 and N205) with up to 16 different types of *N*-glycans identified (155). Most of the IgA *N*-glycans are bi-antennary with the occasional presence of tri- and tetra-antennary *N*-glycans and *N*-glycans of high-mannose types.

The *N*-glycan structures of IgA2 are relatively simple and consist predominantly of high-mannose or bi-antennary types. By contrast, IgA1 exhibits a high degree of sialylation and galactosylation, not only at its *N*-glycosylation sites but also at the *O*-glycosylation sites within the IgA1 hinge region (155, 158).

It is also suggested that IgA2 contains *N*-glycans in the Fab domain of IgA2 (157).

##### O-Glycosylation of IgA

The *O*-glycans in IgA1 contain mainly GalNAc, Gal and sialic acid as a part of the core-1 *O*-glycans. Typically, *O*-glycans of IgA1 are in the form of T-antigen (Gal-GalNAc), sialylated T-antigen or di-sialylated T-antigen (155). Both carbohydrates, i.e. Gal or GalNAc, can be sialylated, forming two different linkages (α2,3-sialic acid or α2,6-sialic acid). The *O*-glycan can also be fucosylated, with the total *O*-glycan length containing 2 to 15 carbohydrates, with more than 50 different glycan structures identified to date (155).

IgA2 does not contain any *O*-glycans present. The content of sialic acid in IgA1 is higher than in IgA2 (106), and such a feature of IgA1 might lead to the lower inflammatory properties of IgA1 in comparison with IgA2 (106).

##### IgA Glycosylation Related to RA

In relation to RA, the main glycosylation change in IgA includes increased levels of bisecting *N*-glycans and sialylation of *O*-glycans, in comparison with HI (159). IgA glycans are not associated with RA disease activity, indicating a different function of IgA in RA from that of IgGs (159).

For example, RA patients exhibit a transition towards pro-inflammatory IgA2 antibodies, which are associated with RA activity. Desialylation enhances the pro-inflammatory capacity of IgA1, rendering it comparable to that of IgA2 (158). Furthermore, reduced GalNAc in *O*-glycans was observed in IgA1 in RA patients, in comparison with HI (155).

IgA2, the more pro-inflammatory IgA subclass and one that is associated with autoimmune diseases, exhibits reduced sialylation, galactosylation, fucosylation, and bisecting glycan structures when compared with IgA1 (157).

#### IgM Glycosylation

Glycoproteomic analysis has revealed the presence of five *N*-glycosylation sites in IgM (N46, N209, N272, N279, and N440) (119). *N*-glycosylation sites N46 and N209 contained predominantly fully galactosylated bi-antennary structures that were core-fucosylated, partially bisected and partially sialylated. Sialylation levels were higher at N209 than at N46. By contrast, N440 exclusively carried oligomannose-type glycans. N272 predominantly carried complex-type glycans characterized by a high degree of bisection, galactosylation and sialylation, with minor glycan compositions indicative of tri-antennary structures. By contrast, N279 was largely decorated with oligomannose-type glycans, accompanied by a substantial proportion of hybrid-type glycans and smaller amounts of complex-type glycans (119).

The changed *N*-glycosylation of IgM in relation to RA showed decreased galactosylation, increased bisecting *N*-glycans and increased fucosylation of *N*-glycans (119).

## Analysis of Glycans as RA Biomarkers

Since changed glycosylation is considered to be a hallmark of RA, it is worth investigating the clinical potential of glycans as RA biomarkers.

### Instrument-Based Analysis of Glycans

There are two main routes for the analysis of glycans present on immunoglobulins.

**Fig. 8:**
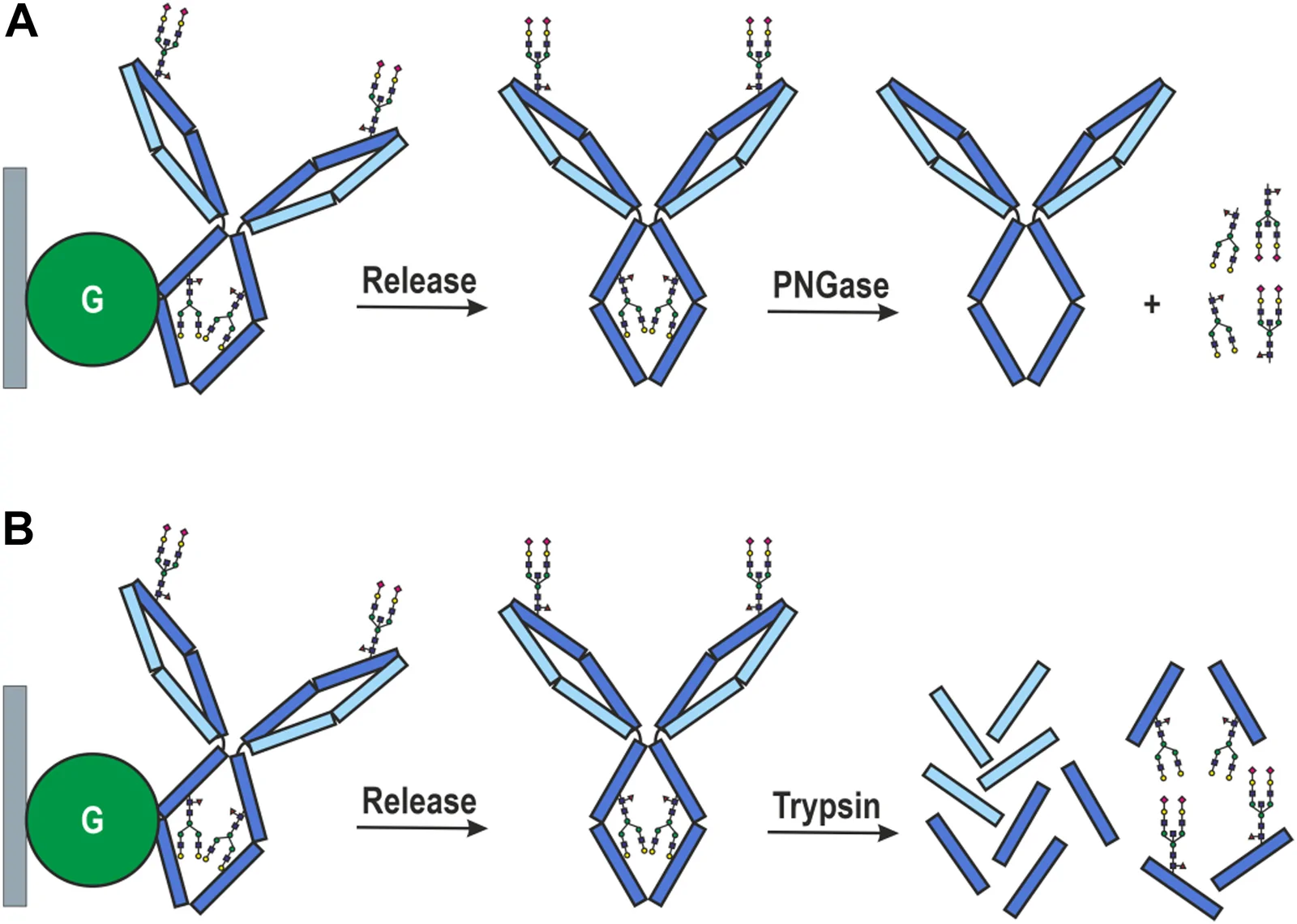
**Instrument-based analysis of*****N*****-glycans released from immunoglobulin G (IgG)**. (*A*) Affinity capture of IgG by immobilized protein G (attached onto beads or plate), followed by release of IgGs from the protein G and then cleavage of *N*-glycans from IgGs by PNGase with a final analysis of *N*-glycans, and (*B*) affinity capture of IgG by immobilized protein G (attached onto beads or plate), followed by release of the IgGs from the protein G and then digestion of IgGs by trypsin with a final analysis of glycopeptides. Other immunoglobulins like IgA can be analyzed in the same way as for IgG as detailed here.

The first route is based on the isolation of IgGs from serum/plasma samples by an affinity interaction with immobilized protein A/G; this is followed by the release of IgGs from the protein A/G and removal of *N*-glycans from IgGs by the enzyme PNGase (peptide:*N*-glycosidase) with a final analysis of *N*-glycans (Fig. 8*A*).

The second route is based on the isolation of IgGs from serum/plasma samples by an affinity interaction with immobilized protein A/G; this is followed by the release of IgGs from the protein A/G and enzymatic digestion of IgGs by the enzyme trypsin with a final analysis of glycopeptides (Fig. 8*B*). With this approach, analysis of *O*-glycopeptides is possible, as also is allocation of the glycan to a particular site of the IgG (i.e. Fc fragment, Fab fragment or hinge fragment).

MALDI Fourier transform ion cyclotron resonance (FTICR) MS was used for analysis of the *O*- and *N*- glycan changes on IgA associated with RA (159). IgA from human serum was enriched based on affinity capture using beads in a 96-well format. The IgA then released was treated with trypsin and analyzed using an instrument-based approach (Fig. 8*B*). The results indicate that increased levels of sialic acid, in particular, within *O*-glycans of IgA1 were significantly higher for RA patients than for HI, and the level of bisecting *N*-glycans at Asn144 was also higher for RA patients than for HI. The study shows only a minor, if any, correlation between disease activity and IgA glycosylation (159).

Hydrophilic interaction liquid chromatography-ultra-performance liquid chromatography was used for analysis of the IgGs glycans in serum samples (160). IgG was isolated from human serum by affinity chromatography using a 96-well protein G monolithic plate. IgG released from the plate was then treated with PNGase to release *N*-glycans (Fig. 8*A*). Subsequent statistical processing identified several significant peaks. The highest discriminatory power was achieved for the GP1 peak, i.e. FA1 glycan, with AUC = 0.881 (160).

Using specialized microfluidic chips, 20 sulfated and 4 acetylated *N*-glycans were detected, many of which were undetectable by other methods due to their low abundance (161). IgG was purified from serum samples by affinity capture using protein A Sepharose beads. IgG eluted from the beads was treated with PNGase to release *N*-glycans (Fig. 8*A*). Then, the *N*-glycans were enriched and analyzed using TiO_2_ and a porous graphitized carbon-based chip. Subsequent analysis of the samples identified several glycan-based biomarkers that could be used to classify positive and negative RA patients for both RF and ACPA with high AUC (Table 6) (161).

**Table 6:** Instrument-Based Methods for Glycan Analysis

Analyte	Glycans	AUC or *p* values	Samples	Ref.
IgA	sialic acid in *O*-glycans	*p* = 0.002 (**SN RA *versus* HI**)^a^	252 RA; 32 HI	(159)
IgA	bisecting *N*-glycan at Asn144	*p* < 0.001 (**SN RA *versus* HI**)	252 RA; 32 HI	(159)
IgG	FA1	AUC = 0.88; *p* < 0.001 (**SN RA *versus* HI**)	128 RA; 195 HI	(160)
IgG	neutral glycans	AUC = 0.85 (RA *versus* HI)	277 RA; 141 HI	(161)
IgG	acidic glycans	AUC = 0.80 (RA *versus* HI)	277 RA; 141 HI	(161)
IgG	sulfated glycans	AUC = 0.86 (RA *versus* HI)	277 RA; 141 HI	(161)
IgG	two sulfo glycans	AUC = 0.88 (RA *versus* HI)	277 RA; 141 HI	(161)
IgG	G0/G1 ratio	AUC = 0.68 (RA *versus* HI)	277 RA; 141 HI	(161)
IgG	G0/(G1+G2∗2)	AUC = 0.90 (RA *versus* HI)	38 RA; 20 HI	(162)
IgG	G0/(G1+G2∗2)	0.91 (SN RA *versus* HI)	38 RA; 20 HI	(162)
IgG2/3, IgA, TSNG	five glycans	AUC = 0.88 (RA *versus* HI)	243 RA; 31 HI	(163)
IgG2/3, IgA, TSNG	five glycans	AUC = 0.88 (**SN RA *versus* HI**)	243 RA; 31 HI	(163)
IgG	3 sialylated glycans	AUC = 0.90 (RA *versus* HI)	105 RA; 79 HI	(164)
IgG	bisecting GlcNAc	*p* = 0.007 (**SN RA *versus* HI**)^b^	12 RA; 10 HI	(169)
IgG1	bisecting GlcNAc	*p* = 0.007 (**SN RA *versus* HI**)^b^	12 RA; 10 HI	(169)
IgG	FA2BG2	*p* = 0.0055 (**SN RA *versus* HI**)^a^	12 SN RA; 11 HI	(171)
IgG	FA2BG2S1	*p* = 0.0051 (**SN RA *versus* HI**)^a^	12 SN RA; 11 HI	(171)
IgG	(G1+G2)/(G0+G1+G2)	*p* < 0.001 (SN *versus* HI)^c^	15 RA; 15 HI	(172)
IgG	low galactose level	*p* = 0.0012 (RA *versus* HI)^c^	44 RA; 30 HI	(173)
IgG	low sialic acid level	*p* < 0.0001 (RA *versus* HI)^c^	44 RA; 30 HI	(173)
IgG	high fucose level	*p* = 0.0063 (RA *versus* HI)^c^	44 RA; 30 HI	(173)

Discrimination SN RA versus HI is shown in **bold**.

TSNG, total serum *N*-glycome.

a Mann–Whitney *U* test.

b Welch *t* test.

c Student *t* test.

Glycan analysis by MALDI-TOF-MS^n^ showed that monitoring of the IgG galactosylation is useful for distinguishing RA from HI (162). IgG was affinity-captured from serum samples using a protein G spin kit. *N*-glycans were released from IgG using PNGase and analyzed by MALDI-TOF-MS^n^ (Fig. 8*A*). An advanced analysis of selected 3 bisecting GlcNAc glycoforms was then performed. The results indicate that, in particular, the level of agalactosylation, i.e. the G0 to (G1+G2∗2) ratio, afforded a very high AUC of 0.90 (sens. 79%; spec. 90%) for discrimination between RA and HI; and an AUC of 0.91 (sens. 82%; spec. 90%) for discrimination between SN RA and HI (162).

One *N*-glycan present in the Fc domain of IgG2/3 (H5N4F1), two *O*-glycans present in IgA (N3H2S1 and N4H4S4) and two glycans detected in the total serum *N*-glycome (TSNG; H5N4F1L1 and H4N5E1) were used as diagnostic RA biomarkers (163). IgG was affinity-captured by using protein G Sepharose beads and then the isolated IgGs were digested with trypsin and the glycopeptides analyzed (Fig. 8*B*). IgA antibodies were purified using affinity matrix beads and the isolated IgA was digested with trypsin and the glycopeptides were analyzed (Fig. 8*B*). TSNG was obtained by release of the *N*-glycans from serum samples using PNGase with *N*-glycans analyzed by MALDI-TOF-MS. The discriminatory power of RA *versus* HI, represented by AUC for all five biomarkers was 0.881. The same AUC of 0.881 was also confirmed for discrimination between SN RA and HI using the same panel of 5 glycan-based biomarkers. Out of the five glycan biomarkers, only two, that is, fucosylated biomarkers IgG2/3 H5N4F1 and TSNG H5N4F1L1, showed significant correlation with DAS28-CRP (163).

Analysis of the glycans on IgGs in serum samples from 105 RA patients and 79 HI showed that three acidic glycans, i.e. bi-antennary glycans containing sialic acid, out of 57 *N*-glycans detected could distinguish RA patients from HI with AUC of 0.90 (164). IgG was purified from serum using a protein A affinity-based isolation step (Fig. 8*A*). Serum IgG glycans were enriched using a TiO_2_ porous graphitic carbon-packed column with the glycan analysis performed using multiple reaction monitoring (164).

**Fig. 9:**
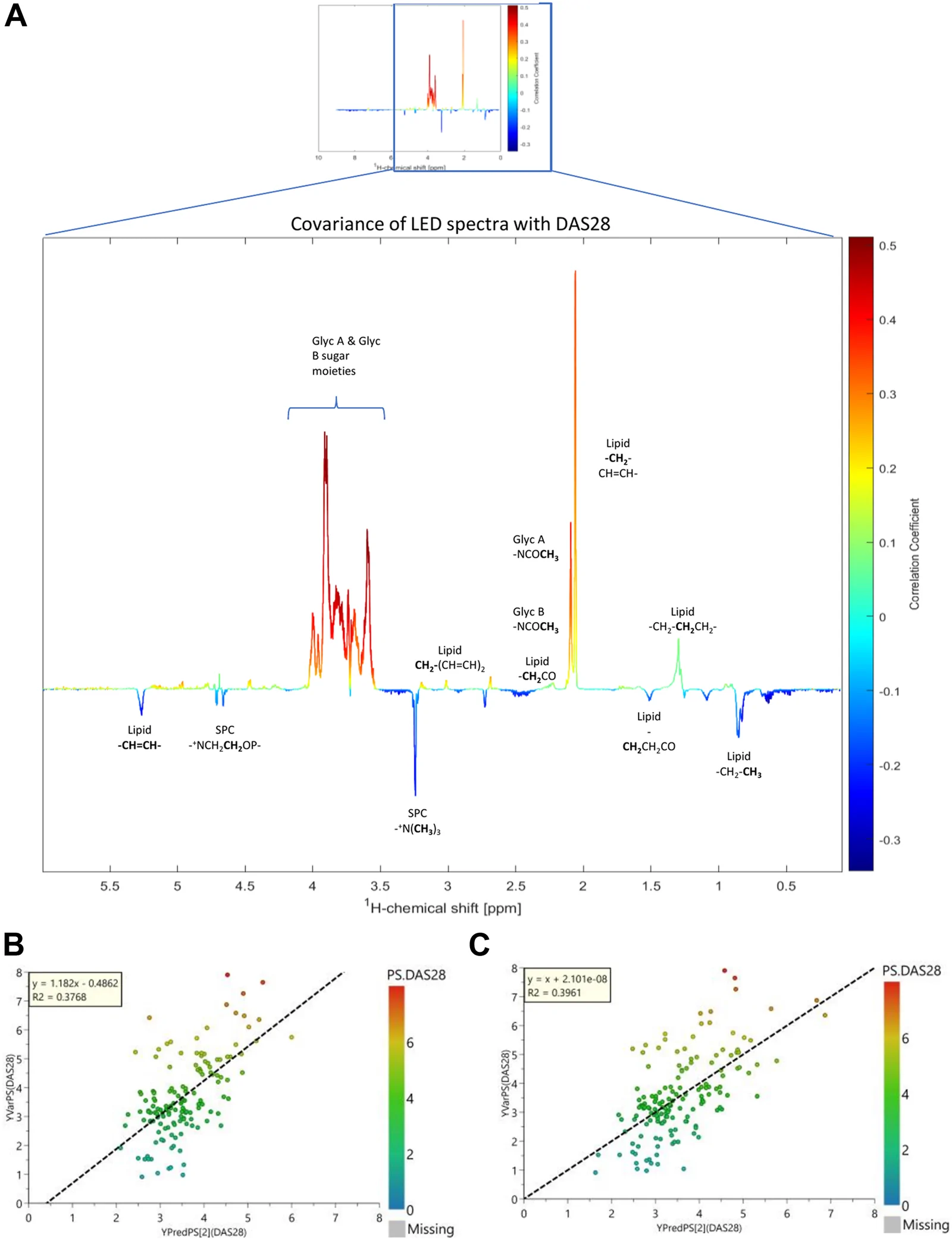
**NMR spectra for reading signals associated with GlycA and GlycB.** Taken from the open access publication (165).

NMR spectra were processed to analyze two *N*-glycoprotein signals GlycA and GlycB (Fig. 9) (165). The results indicate increased levels of GlycA (*N*-acetylglucosamine and *N*-acetylgalactosamine) and GlycB (*N*-acetylneuraminic acid) in RA patients in comparison with HI, showing good discriminatory power with an AUC of 0.90. The GlycA and GlycB levels positively correlated with the DAS28 index and CRP level. Furthermore, GlycA and GlycB could be used as biomarkers for assessing an effective response to therapy (165). The carriers of GlycA and GlycB could be several proteins including *α*_1_-acid glycoprotein, *α*_1_-antichymotrypsin, haptoglobin, *α*1-antitrypsin, and transferrin (166). Another study confirmed a positive correlation between the levels of GlycA and Glyc B with inflammatory markers, underlining their role in the evaluation of RA severity (167).

**Fig. 10:**
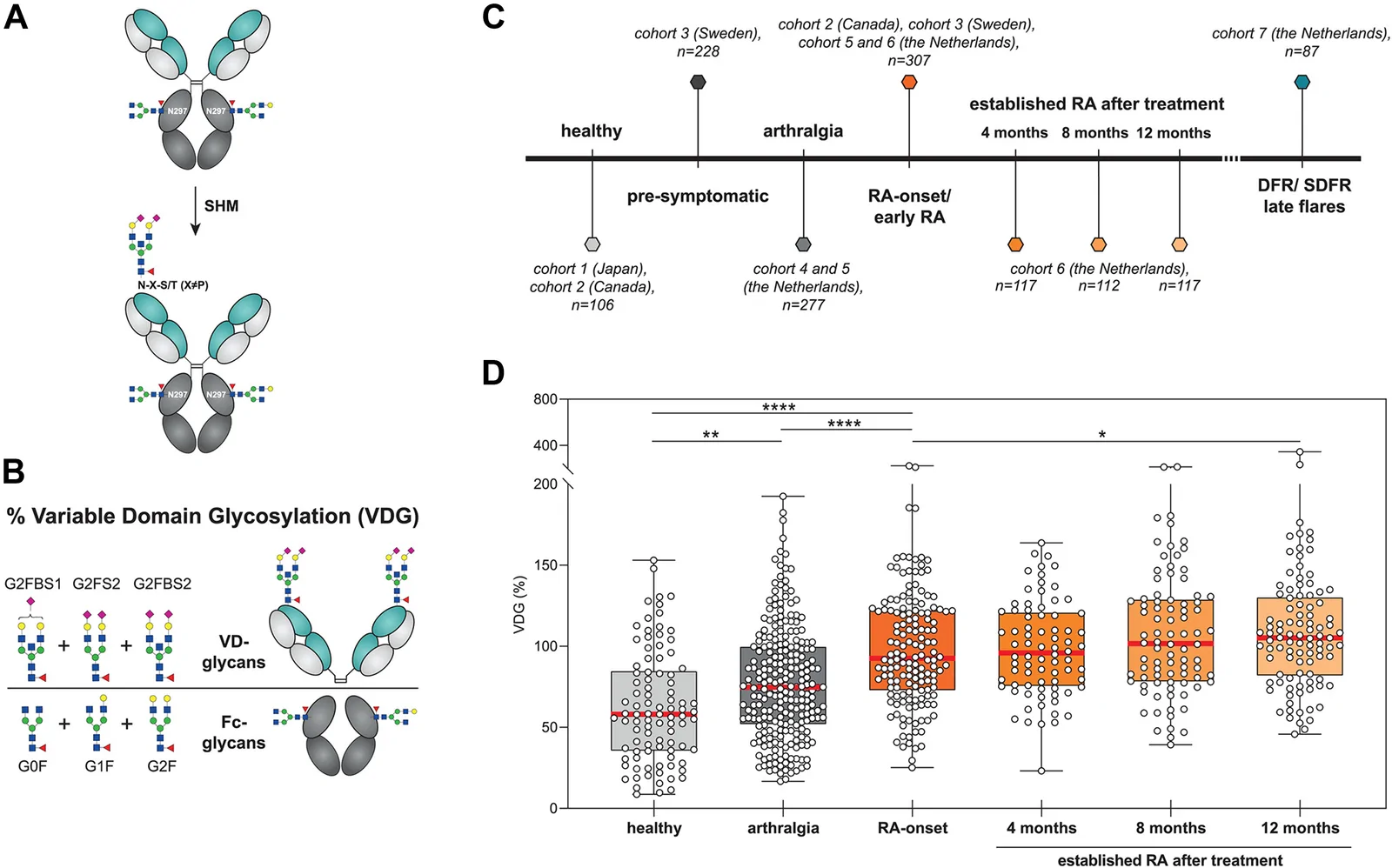
**Percentage of Fab's glycosylation in ACPAs from HI, patients with arthralgia, and RA patients at different disease stages.***A*, IgGs carry *N*-glycans at each N297 residue in the Fc domain and can acquire additional glycosylation sites in the Fab domain by somatic hypermutation. *B*, quantification of *N*-glycosylation in the Fab domain of ACPA, i.e. (sum of the most abundant Fab's glycans/sum of the most abundant Fc glycans) × 100, or [(G2FBS1 + G2FS2 + G2FBS2)/(G0F + G1F + G2F)] × 100, where G0/G1/G2 represents A-/mono-/di-galactosylated, F represents core fucosylated, B represents bisecting *N*-acetylglucosamine (GlcNAc), and S1/S2 represents mono-/di-sialylated. *C*, time course of different RA stages clinical disease stages. *D*, percentage of *N*-glycans in the Fab domain of ACPA in HI, patients with arthralgia, patients at the time of RA onset, and patients with established RA 4 months, 8 months, and 12 months after institution of pre-specified treatment. Data are presented as box and whisker plots and circles represent individual samples. ∗ = *p* = 0.037; ∗∗ = *p* = 0.0032; ∗∗∗∗ = *p* < 0.0001. DFR = drug-free remission; SDRF = sustained drug-free remission. The image was taken from the open access publication (15).

In a very important paper, the changes in IgG ACPA Fabs glycosylation across different clinical RA stages - from healthy, asymptomatic individuals to those with arthralgia (pre-RA phase), and further to patients at disease onset and with established RA, were investigated (Fig. 10) (15). To evaluate the changes in the IgG ACPA Fab glycans, the percentage of IgG ACPA Fabs glycans was quantified using the equation: (sum of the most abundant Fabs glycans/sum of the most abundant Fc glycans) × 100, specifically [(G2FBS1 + G2FS2 + G2FBS2)/(G0F + G1F + G2F)] × 100. Here, G0, G1, and G2 denote agalactosylated, monogalactosylated and digalactosylated structures, respectively; F indicates core fucosylation; B denotes the presence of a bisecting *N*-acetylglucosamine (GlcNAc); and S1 and S2 represent mono- and disialylation, respectively. The results indicate that HI already had Fab glycosylation (56%), which increased in individuals with clinically identified arthralgia (75%), and further increased in the patients with RA onset (93%). Fab glycosylation of ACPA remained fairly stable in RA patients under treatment, with only a moderate increase in glycosylation after 12 months of treatment (105%). Overall, the results indicate that the prevalence of ACPAs' Fab glycosylation is lower in HI and increases during the progression towards RA. However, in already developed RA disease, no further increase in ACPAs IgG glycosylation was observed in the study. The study also confirmed that Fab glycosylation of ACPA was linked with ACPA levels, but also with the ability of ACPA to recognize a wider repertoire of epitopes. The results also indicate that ACPAs Fab glycans are maintained at high levels in established RA and exhibit a slight decrease following immunosuppressive treatment. The findings also indicate that, among individuals who achieve long-term drug-free remission, ACPAs Fab glycosylation is reduced at the time of RA onset (i.e., 61% for RA patients with remission *versus* 84% for RA patients without remission achieved). The study also suggests a direct link between ACPA Fab glycosylation and the main genetic risk factor for RA (i.e., HLA shared epitope alleles). The authors also claimed the following: “Thus it is tempting to speculate that variable domain glycans serve as an additional “hit” determining the fate of the autoreactive B cell response and thereby exert an impact on ACPA levels.” (15).

Serum *N*-glycome analyzed by MALDI-TOF MS was used to distinguish between RA and HI (168). The study revealed a correlation of changed glycosylation with disease activity (DAS28-CRP), which was negative for galactosylated bi-antennary glycans and positive for tri-antennary fucosylated types of glycans. The authors concluded that the protein bearing tri-antennary fucosylated type of glycans was independent of IgG (168).

Comprehensive glycopeptide profiling was performed of IgG, IgA and IgM isolated by affinity-capture from human serum with subsequent MS analysis (119). IgG was affinity-purified by using IgG bead suspension, IgA was affinity-purified by using IgA bead suspension and IgM was affinity-captured by using IgM bead suspension. The isolated immunoglobulins were then treated with trypsin and the glycopeptides released were analyzed using LC-MS. The results showed the following facts when RA patients were compared with HI: 1. decreased galactosylation in Fc *N*-glycan of IgG; 2. decreased galactosylation in IgA2 *N*-glycan; 3. decreased sialylation in IgA1 *O*-glycan; 4. decreased galactosylation in IgM *N*-glycan; 5. increased bisecting IgM *N*-glycan; 6. increased fucosylation of IgM *N*-glycan (119).

A comprehensive study showed that IgA2 had pro-inflammatory properties with a lower content of sialic acid, galactose, fucose and bisecting glycans than IgA1 (158). IgM was isolated from pooled human sera using affinity capture by peptide M agarose. Furthermore, the removal of sialic acids from IgA1 renders it pro-inflammatory and comparable to IgA2. Finally, RA patients display a shift toward IgA2, which is associated with higher RA disease activity (i.e. a positive correlation of IgA2 on ACPA with DAS28) (158).

Paired analysis of IgG subclass galactosylation and sialylation in both ACPA-positive and ACPA-negative IgG demonstrated a moderate inverse correlation with inflammatory markers (CRP, ESR and RF) (169). The IgG was affinity-captured from serum samples using HiTrap protein G affinity purification columns. The ACPA IgG was affinity-purified from IgG fraction using citrulline containing peptide attached to columns. No significant correlation was observed with DAS28 or ACPA levels. A negative correlation between the glycosylation of non-ACPA IgG fractions and DAS28 was observed. The study also highlighted the fact that it was necessary to glycoprofile IgGs in an isoform-specific manner (169).

To run an instrument-based assay, *N*-glycan standards are required. A comprehensive library of all 64 bi-antennary bisecting *N*-glycans was recently achieved and can be used for standardization of *N*-glycan analysis (170).

The key characteristics underlying the analysis of glycans as RA biomarkers using instrument-based approaches are summarised in Table 6 (171, 172, 173).

### Lectin-Based Glycan Analysis

Lectin microarrays exploit the high carbohydrate-binding specificity of lectins, hence are well suited for the comprehensive profiling of glycan patterns in diverse and complex biological samples (174). A lectin microarray using 56 lectins was used for the analysis of human serum samples (9). The human serum samples were incubated with the microarray covered by lectins and finally an anti-human IgG antibody labelled with a fluorescent dye was added to glycoprofile the IgGs present in the samples (9).

In our study, serum samples from 100 individuals, including HI and patients with RA, were analyzed (102). Each sample was evaluated using standard immunoassays to detect 10 RA-associated markers, as well as glycan-profiling of antibodies across 10 different assay formats employing multiple lectins. The resulting dataset was analyzed using artificial neural network-based data-mining approaches. Key RA markers capable of discriminating between HI and SP RA patients (i.e. sera containing autoantibodies) were identified with an accuracy of 83.3%. Notably, integrating RA marker detection with glycan analysis significantly improved the discrimination accuracy to 92.5%. Immunoassays were not able to identify SN RA patients (i.e. sera lacking autoantibodies), whereas glycan analysis correctly classified 43.8% of these cases (102).

**Fig. 11:**
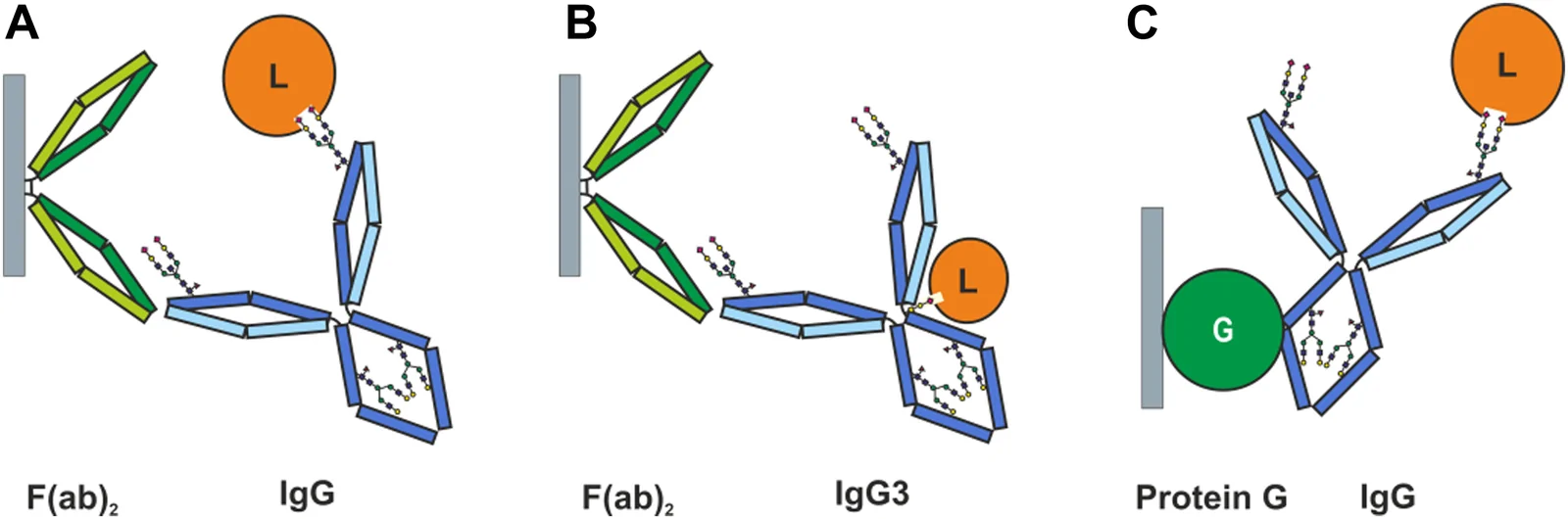
**Affinity-based analysis of glycans on intact IgGs using lectins.** (*A*) affinity capture of IgG by (Fab)2 fragment recognizing Fab fragment of IgG, followed by incubation with lectin (L); (*B*) affinity capture of IgG by (Fab)2 fragment recognizing Fab fragment of IgG3, followed by incubation with lectin (L) also able to recognize O-glycan present in the hinge region of IgG3; (*C*) affinity capture of IgG by protein A/G recognizing Tc domain of IgG, followed by incubation with lectin (L). Other immunoglobulins like IgA can be analyzed in the same way as IgG described here.

A lectin-based and instrument-based approach was used for analysis of IgG glycan changes for discriminating between SP RA and SN RA (171). The results indicate that the lectin LCA (*Lens culinaris* agglutinin) was able to distinguish SP RA patients from SN RA patients with *p* < 0.05. The instrument-based approach proved that, in particular, two fucosylated bisecting IgG glycans, that is, FA2BG2 (bi-antennary digalactosylated glycan) and FA2BG2S1 (bi-antennary digalactosylated monosylated glycan) showed a discriminatory power with p ∼ 0.005. The lectin-based assay was performed in such a way that F(ab)_2_ Fragment Goat Anti-human IgG was immobilized on ELISA plate for specific capture of IgG from the serum samples, and finally the sandwich configuration was completed by incubation with a lectin (171) (Fig. 11*A*).

The effect of B cell depletion therapy for RA patients on the level of sialylation of IgGs and ACPA was investigated (175). The results showed that, while sialylation of IgGs decreased by 14% as a result of the 6-month therapy, the level of sialylation of ACPA remained the same, i.e. elevated compared to HI. The authors used *Sambucus nigra* agglutinin affinity column chromatography for the isolation of the sialylated proteins (IgGs and ACPA) (175).

In addition, one study reported that IgG immune complexes from RA patients exhibited elevated levels of exposed terminal galactose and *N*-acetylgalactosamine (GalNAc) residues on *O*-glycans when compared with HI (176). In the study F(ab)_2_-fragment of goat anti-human IgG, most likely recognizing the Fab fragment of IgG, was immobilized on the surface of ELISA plates (Fig. 11*B*). Then the serum samples from RA patients (i.e. RA patients at baseline and the same RA patients after 3 years of treatment) and HI were incubated with the surface and finally four different lectins were applied, that is, AAL (recognizing fucose), LCA (detecting core fucosylated tri-mannosylated glycans), SNA (recognising α2,6-linked sialic acid) and Jacalin (detecting core 1 and 3 *O*-glycans terminated in Gal, GalNAc and GlcNAc). The results indicate that IgG from RA patients formed IgG immunocomplexes with several proteins (i.e., IgM, IgA, C1q, C3c, CRP) more frequently than with IgG from HI, suggesting a change in the native structure of IgG in RA patients compared with HI. Furthermore, the level of fucose on the IgG immunocomplexes was significantly higher in RA patients than in HI and correlated positively with ACPA. A significantly higher signal for SNA and Jacalin was observed on IgG immunocomplexes for RA patients at baseline. While the SNA response increased for RA-treated patients, the Jacalin response decreased for RA-treated patients with an increase in the response of SNA-to-Jacalin ratio indicating a successful glucocorticoid treatment. It is not clear whether the study described, for the first time, the presence of *O*-glycans on IgG (IgG3) or whether *O*-glycans were present on other proteins forming complexes with IgG (i.e. IgM, IgA, C1q, C3c, CRP) (176).

The changed glycosylation of clusterin could be used as an RA biomarker (177). The polyclonal antibody against clusterin was adsorbed on the ELISA plates and, after incubation with human serum samples, 5 different biotinylated lectins recognizing either sialic acid (MAA and SNA) and fucose (LTA, UEA and LCA) were used for the glycoprofiling of clusterin. The results showed that the glycoprofiling of clusterin by SNA could provide discriminatory power for RA *versus* HI with an AUC of 0.735 (177).

A lectin microarray was used for the incubation with human serum samples from RA patients and HI (178). In order to detect glycans only on metalloproteinase-3, the lectin microarray was finally incubated with an antibody against metalloproteinase-3. Thereby, it was possible to glycoprofile metalloproteinase-3. An intensive signal was observed using four different lectins, but the authors decided to calculate the ratio of two lectins, i.e. ACG/Jacalin score (ACG = *Agrocybe cylindracea* galectin). The results indicate a correlation of ACG/Jacalin score (i.e. degree of *O*-glycan 2,6-sialylation) with the disease activity index and also with a change in the disease activity index associated with RA treatment (178).

The microtiter plate was functionalized with a protein A layer to selectively capture IgG molecules from human serum via their Fc domains, thereby orientating the antibodies with the Fab fragments exposed to the solution. This enzyme-linked lectin assay (ELLA) format enabled specific glycoprofiling of IgG Fab fragments (Fig. 11*C*). The results demonstrated that the ELLA format could distinguish RA patients from HI with an AUC of 0.917, and SN RA patients from HI with an AUC of 0.904 using only three lectins. Additionally, significant differences in Fab-associated glycan profiles were observed between the SN and SP cohorts. Notably, the findings also suggest the potential presence of *O*-linked glycans within the Fab region of IgG molecules (data not shown).

The key characteristics underlying the analysis of glycans as RA biomarkers using lectin-based approaches are summarized in Table 7 (179).

**Table 7:** Lectin-based methods for glycan analysis

Analyte	Lectin/glycan	AUC or *p* values	Samples	Ref.
IgG	SBA	0.65 (RA *versus* HI)	214 RA; 100 HI	(9)
ACPA, RF and RCA ELISA of IgGs	Various lectins	0.650–0.860 (RA *versus* HI)	47 RA; 53 HI	(102)
IgG	LCA	*p* < 0.05 (**SN RA *versus* HI**)^a^	12 SN RA; 11 HI	(171)
IgG	AAL	*p* = 0.002 (**SN RA *versus* HI**)^b^	30 RA; 30 HI	(176)
IgG	LCA	*p* = 0.001 (**SN RA *versus* HI**)^b^	30 RA; 30 HI	(176)
IgG	SNA	*p* < 0.0001 (**SN RA *versus* HI**)^b^	30 RA; 30 HI	(176)
IgG	Jacalin	*p* = 0.002 (**SN RA *versus* HI**)^b^	30 RA; 30 HI	(176)
Clusterin	SNA	0.73 (RA *versus* HI)	34 RA; 21 HI	(177)
IgG	DSA	*p* < 0.001 (SN *versus* HI)^a^	40 RA; 20 HI	(179)
IgG	SNA	*p* < 0.05 (SN *versus* HI)^a^	40 RA; 20 HI	(179)
IgG	MAA	*p* < 0.001 (SN *versus* HI)^a^	40 RA; 20 HI	(179)
IgG	AAL	*p* < 0.05 (SN *versus* HI)^a^	40 RA; 20 HI	(179)
IgG	MAA + DBA + PHAL	0.917 (RA *versus* HI)^a^	84 RA; 40 HI	Unpublished work
IgG	MALA + GSL_I + AAL	0.904 (**SN RA *versus* HI**)	84 RA; 40 HI	Unpublished work

Discrimination SN RA versus HI is shown in **bold**.

AAL, *Aleuria aurantia* lectin; DBA, *Dolichos biflorus* agglutinin; DSA, *Datura stramonium* agglutinin; GSL_I, *Griffonia (Bandeiraea) simplicifolia* lectin; LCA, *Lens culinaris* agglutinin; MAA, *Maackia amurensis* agglutinin; PHAL, *Phaseolus vulgaris* leucoagglutinin; RCA, *Ricinus communis* agglutinin; SBA, soybean agglutinin; SNA, *Sambucus nigra* agglutinin.

a Mann-Whitney U test.

b Welch *t* test.

## Conclusion

This review article provides evidence that novel diagnostic RA biomarkers and, in particular, biomarkers for diagnostics of seronegative RA are still required, and that a substantial effort has been devoted to identifying prospective novel biomarkers in recent years. However, in order to prove their potential for diagnostics of SN RA, large clinical validation studies are required. Among them, glycan-based biomarkers have exhibited a very promising clinical performance. For example, the instrument-based approaches analyzing glycans present on immunoglobulins showed AUC up to 0.90 (RA *versus*. HI) or 0.91 (SN RA *versus* HI). The use of lectins for affinity-based analysis of glycans present on immunoglobulins showed a very similar clinical performance with AUC of 0.92 (RA *versus* HI) and 0.90 (SN RA *versus* HI).

When these two different approaches underlying the analysis of glycans on immunoglobulins are compared, the affinity-based glycan assays present significant advantages: 1. Analysis of glycans using lectins can be performed on intact immunoglobulins; 2. Integration of the step to capture immunoglobulins using protein A or G layers makes it possible to attach immunoglobulins onto surfaces so that the glycans present in the Fab fragment or in the hinge region of IgG3 can be accessible for lectin-binding, making it possible to specifically target the glycan in these two domains; 3. In the case that glycans present in the Fc domain of immunoglobulins are targeted, these immunoglobulins can be captured on the surface using antigens, that is, cyclic citrullinated peptides.

There are two different lectin-based approaches that can be used for the analysis of glycans on immunoglobulins, that is, lectin microarrays and ELISA-like lectin assays. While lectin microarrays can offer high-throughput analysis, ELISA-like lectin assays are analytically more sensitive.

Part of the workflow underlying instrument-based approaches can be performed in an ELISA-like format. For example, the GlYcoLISA combines a conventional 96-well-based immunosorbent assay with LC-MS profiling of IgG Fc *N*-glycosylation in a high-sensitivity, throughput-optimized manner (180). Lectin-based methods entailed in glycan analysis can be fully performed in the ELISA assay format from the beginning to signal generation. Furthermore, ELISA-like assay formats can be readily transferred onto the automatic machines of industry leaders.

It is also very important to realise that analysis performed in serum/plasma samples can be different from the analysis performed in the synovial fluid. Notably, distinct IgG glycan structures were identified in the study (181). The bi-antennary, bisected, core-fucosylated IgG glycan reported in other studies was not detected in the analysis. By contrast, a novel antennary fucosylated, tri-antennary glycan structure on IgG1 exhibited a significant difference between RA and osteoarthritis (OA) samples. The authors explain these results by the fact that, in the previous studies, serum samples were used, unlike in the current study where synovial fluid was used, indicating that the source of the samples had a significant impact on the glycosylation profiles (181). Hafkenscheid *et al.* showed the level of Fab glycosylation on ACPA to be higher when the synovial fluids assays were compared with plasma samples, while the level of Fab glycosylation of total IgG was approximately the same in plasma samples and synovial fluids (146). Furthermore, the Fc glycosylation of ACPA isolated from synovial fluids contained more pro-inflammatory Fc *N*-glycans than the ACPA isolated from serum (146).

## Data Availability

All data generated or analyzed during this study are included in this published article and its supporting information files. Raw data is available upon request.

## Conflict of Interest

The authors declare that they have no conflicts of interest with the contents of this article.

## References

[1] Xue M., Wang H., Campos F., Jackson C.J., March L. (2025). Rheumatoid arthritis: biomarkers and the latest breakthroughs. Int. J. Mol. Sci..

[2] Sahin D., Di Matteo A., Emery P. (2025). Biomarkers in the diagnosis, prognosis and management of rheumatoid arthritis: a comprehensive review. Ann. Clin. Biochem..

[3] Qi X.-Y., Liu M.-X., Jiang X.-J., Gao T., Xu G.-Q., Zhang H.-Y. (2025). Gut microbiota in rheumatoid arthritis: mechanistic insights, clinical biomarkers, and translational perspectives. Autoimmun. Rev..

[4] Sadeghi M., Afshari J.T., Fadaee A., Dashti M., Kheradmand F., Dehnavi S. (2025). Exosomal miRNAs involvement in pathogenesis, diagnosis, and treatment of rheumatoid arthritis. Heliyon.

[5] Srivastava S., Rasool M. (2024). Genetics, epigenetics and autoimmunity constitute a Bermuda triangle for the pathogenesis of rheumatoid arthritis. Life Sci..

[6] Hitchon C.A., El-Gabalawy H.S. (2025). Advances in understanding preclinical rheumatoid arthritis and prospects for prevention: Rheumatoid arthritis in 2025. Nat. Rev. Rheumatol..

[7] Di Matteo A., Bathon J.M., Emery P. (2023). Rheumatoid arthritis. Lancet.

[8] Black R.J., Cross M., Haile L.M., Culbreth G.T., Steinmetz J.D., Hagins H. (2023). Global, regional, and national burden of rheumatoid arthritis, 1990–2020, and projections to 2050: a systematic analysis of the global burden of disease study 2021. Lancet Rheumatol..

[9] Deng X., Liu X., Zhang Y., Ke D., Yan R., Wang Q. (2023). Changes of serum IgG glycosylation patterns in rheumatoid arthritis. Clin. Proteomics.

[10] Uke P., Maharaj A., Adebajo A. (2025). A review on the epidemiology of rheumatoid arthritis: an update and trends from current literature. Best Pract. Res. Clin. Rheumatol..

[11] Taylor P.C. (2023). Pain in the joints and beyond; the challenge of rheumatoid arthritis. Lancet Rheumatol..

[12] Perera J., Delrosso C.A., Nerviani A., Pitzalis C. (2024). Clinical phenotypes, serological biomarkers, and synovial features defining seropositive and seronegative rheumatoid arthritis: a literature review. Cells.

[13] Jiang X., Frisell T., Askling J., Karlson E.W., Klareskog L., Alfredsson L. (2015). To what extent is the familial risk of rheumatoid arthritis explained by established rheumatoid arthritis risk factors?. Arthritis Rheumatol..

[14] Frisell T., Hellgren K., Alfredsson L., Raychaudhuri S., Klareskog L., Askling J. (2016). Familial aggregation of arthritis-related diseases in seropositive and seronegative rheumatoid arthritis: a register-based case-control study in Sweden. Ann. Rheum. Dis..

[15] Kissel T., Hafkenscheid L., Wesemael T.J., Tamai M., Kawashiri S.-Y., Kawakami A. (2022). IgG anti–citrullinated protein antibody variable domain glycosylation increases before the onset of rheumatoid arthritis and stabilizes thereafter: a cross-sectional study encompassing ∼1,500 samples. Arthritis Rheumatol..

[16] Blöchl C., Stork E.M., Scherer H.U., Toes R.E., Wuhrer M., Domínguez-Vega E. (2025). Fc proteoforms of ACPA IgG discriminate autoimmune responses in plasma and synovial fluid of rheumatoid arthritis patients and associate with disease activity. Adv. Sci..

[17] Gravallese E.M., Firestein G.S. (2023). Rheumatoid arthritis—common origins, divergent mechanisms. New Engl. J. Med..

[18] Kitas G.D., Gabrie S.E. (2011). Cardiovascular disease in rheumatoid arthritis: state of the art and future perspectives. Ann. Rheum. Dis..

[19] Listing J., Kekow J., Manger B., Burmester G.R., Pattloch D., Zink A. (2015). Mortality in rheumatoid arthritis: the impact of disease activity, treatment with glucocorticoids, TNFα inhibitors and rituximab. Ann. Rheum. Dis..

[20] Pamies A., Vallvé J.C., Paredes S. (2025). New cardiovascular risk biomarkers in rheumatoid arthritis: implications and clinical utility—A narrative review. Biomedicines.

[21] Kim S.C., Schneeweiss S., Liu J., Solomon D.H. (2013). The risk of venous thromboembolism in patients with rheumatoid arthritis. Arthritis Care Res..

[22] Al-Ewaidat O.A., Naffaa M.M. (2024). Stroke risk in rheumatoid arthritis patients: exploring connections and implications for patient care. Clin. Exp. Med..

[23] Brooks R.T., England B.R., Kronzer V.L., Crowson C.S. (2026). The evolving comorbidity landscape of rheumatoid arthritis. Nat. Rev. Rheumatol..

[24] Jahid M., Khan K.U., Ahmed R.S. (2023). Overview of rheumatoid arthritis and scientific understanding of the disease. Mediterr. J. Rheumatol..

[25] Li D.P., Han Y.X., He Y.S., Wen Y., Liu Y.C., Fu Z.Y. (2023). A global assessment of incidence trends of autoimmune diseases from 1990 to 2019 and predicted changes to 2040. Autoimmun. Rev..

[26] Pierdominici M., Borgi M., Spinelli F.R., Conti F., Wu H., Moon K.W. (2026). Sex and gender differences in rheumatology: clinical impact and future directions. Nat. Rev. Rheumatol..

[27] Kastbom A., Turcinov S., Malmström V. (2025). Windows for intervention in the prearthritis phase of rheumatoid arthritis? A narrative review of key triggering events and potential preventive strategies. RMD Open.

[28] Kumar A., Singh S., Goel F., Pandey R.K., Singh L., Kumar A. (2025). Molecular mechanisms and risk factors in rheumatoid arthritis: a comprehensive review. Inflammopharmacology.

[29] Brewer R.C., Lanz T.V., Hale C.R., Sepich-Poore G.D., Martino C., Swafford A.D. (2023). Oral mucosal breaks trigger anti-citrullinated bacterial and human protein antibody responses in rheumatoid arthritis. Sci. transl. Med..

[30] Steiner G., Toes R.E. (2024). Autoantibodies in rheumatoid arthritis–rheumatoid factor, anticitrullinated protein antibodies and beyond. Curr. Opin. Rheumatol..

[31] Smolen J.S., Aletaha D., Barton A., Burmester G.R., Emery P., Firestein G.S. (2018). Rheumatoid arthritis. Nat. Rev. Dis. Primers.

[32] Roberson E.D.O., Bowcock A.M. (2010). Psoriasis genetics: breaking the barrier. Trends Genet..

[33] Böhler C., Radner H., Smolen J.S., Aletaha D. (2013). Serological changes in the course of traditional and biological disease modifying therapy of rheumatoid arthritis. Ann. Rheum. Dis..

[34] Waaler E. (1940). On the occurrence of a factor in human serum activating the specific agglutination of sheep blood corpuscles. APMIS.

[35] Arnold J.N., Wormald M.R., Sim R.B., Rudd P.M., Dwek R.A. (2007). The impact of glycosylation on the biological function and structure of human immunoglobulins. Annu. Rev. Immunol..

[36] Yi Y., Lei L., Sun Y., Mei J., Zhang Y., Chen J. (2025). Biomarkers for early diagnosis of rheumatoid arthritis. Clin. Chim. Acta.

[37] Soyfoo M., Sarrand J. (2026). Biomarkers in rheumatoid arthritis: from traditional serology to precision medicine integration. Diagnostics.

[38] Haro I., Sanmartí R., Gómara M.J. (2022). Implications of post-translational modifications in autoimmunity with emphasis on citrullination, homocitrullination and acetylation for the pathogenesis, diagnosis and prognosis of rheumatoid arthritis. Int. J. Mol. Sci..

[39] Christophorou M.A. (2022). The virtues and vices of protein citrullination. R. Soc. Open Sci..

[40] Van Steendam K., Tilleman K., Deforce D. (2011). The relevance of citrullinated vimentin in the production of antibodies against citrullinated proteins and the pathogenesis of rheumatoid arthritis. Rheumatology.

[41] Turesson C., Rönnelid J., Kastbom A. (2025). Autoantibodies as prognostic markers in rheumatoid arthritis. J. Transl. Autoimmun..

[42] Reed E., Hedström A.K., Hansson M., Mathsson-Alm L., Brynedal B., Saevarsdottir S. (2020). Presence of autoantibodies in “seronegative” rheumatoid arthritis associates with classical risk factors and high disease activity. Arthritis Res. Ther..

[43] Sieghart D., Konrad C., Swiniarski S., Haslacher H., Aletaha D., Steiner G. (2023). The diagnostic and prognostic value of IgG and IgA anti-citrullinated protein antibodies in patients with early rheumatoid arthritis. Front. Immunol..

[44] Rönnelid J., Hansson M., Mathsson-Alm L., Cornillet M., Reed E., Jakobsson P.-J. (2018). Anticitrullinated protein/peptide antibody multiplexing defines an extended group of ACPA-positive rheumatoid arthritis patients with distinct genetic and environmental determinants. Ann. Rheum. Dis..

[45] Sieghart D., Platzer A., Studenic P., Alasti F., Grundhuber M., Swiniarski S. (2018). Determination of autoantibody isotypes increases the sensitivity of serodiagnostics in rheumatoid arthritis. Front. Immunol..

[46] Li K., Mo W., Wu L., Wu X., Luo C., Xiao X. (2021). Novel autoantibodies identified in ACPA-negative rheumatoid arthritis. Ann. Rheum. Dis..

[47] van Delft M.A.M., Verheul M.K., Burgers L.E., Derksen V.F.A.M., van der Helm-van Mil A.H.M., van der Woude D. (2017). The isotype and IgG subclass distribution of anti-carbamylated protein antibodies in rheumatoid arthritis patients. Arthritis Res. Ther..

[48] Jin Y., Liu J., Desai R.J., Kim S.C. (2023). Real-World treatment effectiveness of disease-modifying antirheumatic drugs by serostatus among patients with rheumatoid arthritis. ACR Open Rheumatol..

[49] Janssen K.M., de Smit M.J., Brouwer E., de Kok F.A., Kraan J., Altenburg J. (2015). Rheumatoid arthritis–associated autoantibodies in non–rheumatoid arthritis patients with mucosal inflammation: a case–control study. Arthritis Res. Ther..

[50] Ponikowska M., Świerkot J., Nowak B., Korman L., Wiland P. (2019). Autoantibody and metalloproteinase activity in early arthritis. Clin. Rheumatol..

[51] Sidiras P., Lechanteur J., Imbault V., Sokolova T., Durez P., Gangji V.D.S.R. (2022). Human carbamylome description identifies carbamylated α2-macroglobulin and hemopexin as two novel autoantigens in early rheumatoid arthritis. Rheumatology (Oxford).

[52] Saito M., Hess D., Eglinger J., Fritsch A.W., Kreysing M., Weinert B.T. (2019). Acetylation of intrinsically disordered regions regulates phase separation. Nat. Chem. Biol..

[53] Wen J., Zhang Y., Lu Y., Chen X., Zhu L., Zhang G. (2026). Acetylation in autoimmune diseases: from mechanistic insights to therapeutic prospects. Biochem. Pharmacol..

[54] Harris M.L., Darrah E., Lam G.K., Bartlett S.J., Giles J.T., Grant A.V. (2008). Association of autoimmunity to peptidyl arginine deiminase type 4 with genotype and disease severity in rheumatoid arthritis. Arthritis Rheum..

[55] Moore R.E., Wang T., Duvvuri B., Feser M.L., Deane K.D., Solomon J.J. (2023). Prediction of erosive disease development by antimitochondrial antibodies in rheumatoid arthritis. Arthritis Rheumatol..

[56] Thiele G.M., Duryee M.J., Anderson D.R., Klassen L.W., Mohring S.M., Young K.A. (2015). Malondialdehyde-acetaldehyde adducts and anti-malondialdehyde-acetaldehyde antibodies in rheumatoid arthritis. Arthritis Rheumatol..

[57] Nikolov A., Blazhev A., Tzekova M., Kostov K., Popovski N. (2020). Serum levels of antibodies to advanced glycation end products in patients with type 2 diabetes mellitus and hypertension. Folia Med.(Plovdiv).

[58] van den Beukel M.D., van Wesemael T.J., Hoogslag A.T.W., Borggreven N.V., Huizinga T.W.J., van der Helm-Van Mil A.H.M. (2023). Antibodies against advanced glycation end-products and malondialdehyde-acetaldehyde adducts identify a new specific subgroup of hitherto patients with seronegative arthritis with a distinct clinical phenotype and an HLA class II association. RMD Open.

[59] Cappelli L.C., Gutierrez A.K., Bingham C.O., Shah A.A. (2017). Rheumatic and musculoskeletal immune-related adverse events due to immune checkpoint inhibitors: a systematic review of the literature. Arthritis Care Res..

[60] Ghosh N., Tiongson M.D., Stewart C., Chan K.K., Jivanelli B., Cappelli L. (2021). Checkpoint inhibitor-associated arthritis: a systematic review of case reports and case series. J. Clin. Rheumatol..

[61] Cappelli L.C., Bingham C.O., Forde P.M., Anagnostou V., Brahmer J., Lipson E.J. (2022). Anti-RA33 antibodies are present in a subset of patients with immune checkpoint inhibitor-induced inflammatory arthritis. RMD Open.

[62] Neto M., Mendes B., Albuquerque F., da Silva J.A.P. (2025). Novel biomarkers in RA: implication for diagnosis, prognosis, and personalised treatment. Best Pract. Res. Clin. Rheumatol..

[63] Boutet M.A., Nerviani A., Lliso-Ribera G., Leone R., Sironi M., Hands R. (2021). Circulating and synovial Pentraxin-3 (PTX3) expression levels correlate with Rheumatoid arthritis severity and tissue infiltration independently of conventional treatments response. Front. Immunol..

[64] Di Lorenzo B., Zoroddu S., Mangoni A.A., Sotgia S., Paliogiannis P., Erre G.L. (2024). Association between blood Pentraxin-3 concentrations and rheumatic diseases: a systematic review and meta-analysis. Eur. J. Clin. Invest..

[65] Kalka M., Ptak J., Gregorczyk P., Ciura K., Chorążewska A., Haldar S. (2025). Dissecting the specificity of sugar code recognition - unleashing the biomedical potential of galectins by protein engineering. Biotechnol. Adv..

[66] Krstić N., Jevtović Stoimenov T., Stojanović S., Đorđević B., Damnjanović I. (2025). Galectin-1 as a potential biomarker of rheumatoid arthritis drug effectiveness. Int. J. Immunopathol. Pharmacol..

[67] Triguero-Martínez A., de la Fuente H., Montes N., Ortiz A.M., Roy-Vallejo E., Castañeda S. (2020). Validation of galectin-1 as potential diagnostic biomarker of early rheumatoid arthritis. Sci. Rep..

[68] Maged L.A., Saeed S., Abdelfattah W., Gaber W. (2023). Clinical significance of Galectin-1 and Galectin-4 in rheumatoid arthritis patients and their potential role as diagnostic markers. Egypt. Rheumatol..

[69] Mendez-Huergo S.P., Hockl P.F., Stupirski J.C., Maller S.M., Morosi L.G., Pinto N.A. (2019). Clinical relevance of galectin-1 and galectin-3 in rheumatoid arthritis patients: differential regulation and correlation with disease activity. Front. Immunol..

[70] Mahalleh M., Behnoush A.H., Khalaji A., Gouravani M., Assempoor R., Jamshidi A. (2024). Serum Galectin-3 level in patients with rheumatoid arthritis: a systematic review and meta-analysis. Iran. J. Allergy Asthma Immunol..

[71] Nielsen M., Køster D., Greisen S., Troldborg A., Stengaard-Pedersen K., Junker P. (2023). Increased synovial galectin-3 induce inflammatory fibroblast activation and osteoclastogenesis in patients with rheumatoid arthritis. Scand. J. Rheumatol..

[72] Nielsen M.A., Køster D., Mehta A.Y., Stengaard-Pedersen K., Busson P., Junker P. (2023). Increased galectin-9 levels correlate with disease activity in patients with DMARD-naïve rheumatoid arthritis and modulate the secretion of MCP-1 and IL-6 from synovial fibroblasts. Cells.

[73] Zou L., Fan Q., Liu Y., He H., Jia C. (2025). Leucine-rich alpha-2-glycoprotein 1 (LRG1) decrement during biologics therapy and its correlation with disease features and treatment outcomes in rheumatoid arthritis patients. Clin. Rheumatol..

[74] Fujimoto M., Hosono Y., Serada S., Suzuki Y., Ohkawara T., Murata O. (2024). Leucine-rich α2-glycoprotein as a useful biomarker for evaluating disease activity in rheumatoid arthritis. Mod. Rheumatol..

[75] Wu Y., Yang M., Xu Y., Jia L., Li J., Wang X. (2026). A comprehensive N-glycoproteome atlas reveals tissue-specific glycan remodeling but non-random structural microheterogeneities. Nat. Commun..

[76] Di Lorenzo B., Zoroddu S., Mangoni A.A., Paliogiannis P., Erre G.L., Carru C. (2025). Circulating Fetuin-A concentrations in rheumatic diseases: a systematic review and meta-analysis. Eur. J. Clin. Invest..

[77] Pàmies A., Llop D., Ibarretxe D., Rosales R., Girona J., Masana L. (2024). Enhanced association of novel cardiovascular biomarkers Fetuin-A and catestatin with serological and inflammatory markers in rheumatoid arthritis patients. Int. J. Mol. Sci..

[78] Xie Y., Wei C., Fu D., Zhang W., Du Y., Huang C. (2025). Large-scale multicenter study reveals anticitrullinated SR-A peptide antibody as a biomarker and exacerbator for rheumatoid arthritis. Sci. Adv..

[79] Sonuç Karaboğa M.N., Okuyan H.M., Dogan S., Oguzman H., Kimyon G. (2025). Association of UCMA with cartilage pathogenesis and inflammation in patients with rheumatoid arthritis: a novel biomarker. J. Clin. Lab. Anal..

[80] Dominic S., Baba K.S.S.S., Sreedevi N.N., Sanober A., Rajasekhar L., Khan S.A. (2024). Clinical utility of pro-inflammatory oligomeric glycoprotein Tenascin-C in the diagnosis of seropositive and seronegative rheumatoid arthritis. Indian J. Clin. Biochem..

[81] Liang Z., Wang N., Shang L., Wang Y., Feng M., Liu G. (2022). Evaluation of the immune feature of ACPA-negative rheumatoid arthritis and the clinical value of matrix metalloproteinase-3. Front. Immunol..

[82] Vrablova V., Kosutova N., Blsakova A., Bertokova A., Kasak P., Bertok T. (2023). Glycosylation in extracellular vesicles: isolation, characterization, composition, analysis and clinical applications. Biotechnol. Adv..

[83] Brenis Gómez C.M., Plasencia-Rodríguez C., Novella-Navarro M., Martínez-Feito A., Balsa A., Calvo-Aranda E. (2025). Exosomal protein biomarkers in arthritis: deciphering the inflammatory profiles of RA and OA. Biomedicines.

[84] Yoo J., Lee S.K., Lim M., Sheen D., Choi E.-H., Kim S.A. (2017). Exosomal amyloid A and lymphatic vessel endothelial hyaluronic acid receptor-1 proteins are associated with disease activity in rheumatoid arthritis. Arthritis Res. Ther..

[85] Rydland A., Heinicke F., Nyman T.A., Trøseid A.-M.S., Flåm S.T., Stensland M. (2025). Newly-diagnosed rheumatoid arthritis patients have elevated levels of plasma extracellular vesicles with protein cargo altered towards inflammatory processes. Sci. Rep..

[86] Ucci F.M., Recalchi S., Barbati C., Manganelli V., Capozzi A., Riitano G. (2022). Citrullinated and carbamylated proteins in extracellular microvesicles from plasma of patients with rheumatoid arthritis. Rheumatology.

[87] Qin Q., Song R., Du P., Gao C., Yao Q., Zhang J.-A. (2021). Systemic proteomic analysis reveals distinct exosomal protein profiles in rheumatoid arthritis. J. Immunol. Res..

[88] Rodriguez-Jimenez N.A., Gonzalez-Ponce F., Gamez-Nava J.I., Ramirez-Villafaña M., Saldaña-Cruz A.M., Ponce-Guarneros J.M. (2024). Syndecan-1 levels in females with active rheumatoid arthritis. J. Clin. Med..

[89] Centola M., Cavet G., Shen Y., Ramanujan S., Knowlton N., Swan K.A. (2013). Development of a multi-biomarker disease activity test for rheumatoid arthritis. PLoS One.

[90] Hirata S., Dirven L., Shen Y., Centola M., Cavet G., Lems W.F. (2013). A multi-biomarker score measures rheumatoid arthritis disease activity in the BeSt study. Rheumatology.

[91] Meznerics F.A., Kemeny L.V., Gunther E., Bako E., Dembrovszky F., Szabo B. (2023). Multibiomarker disease activity score: an objective tool for monitoring rheumatoid arthritis? A systematic review and meta-analysis. Rheumatology.

[92] Curtis J.R., Weinblatt M.E., Shadick N.A., Brahe C.H., Østergaard M., Hetland M.L. (2021). Validation of the adjusted multi-biomarker disease activity score as a prognostic test for radiographic progression in rheumatoid arthritis: a combined analysis of multiple studies. Arthritis Res. Ther..

[93] Momtazmanesh S., Nowroozi A., Rezaei N. (2022). Artificial intelligence in rheumatoid arthritis: current status and future perspectives: a state-of-the-art review. Rheumatol. Ther..

[94] Danieli M.G., Brunetto S., Gammeri L., Palmeri D., Claudi I., Shoenfeld Y. (2024). Machine learning application in autoimmune diseases: state of art and future prospectives. Autoimmun. Rev..

[95] Kingsmore K.M., Puglisi C.E., Grammer A.C., Lipsky P.E. (2021). An introduction to machine learning and analysis of its use in rheumatic diseases. Nat. Rev. Rheumatol..

[96] Wang J., Tian Y., Zhou T., Tong D., Ma J., Li J. (2023). A survey of artificial intelligence in rheumatoid arthritis. Rheumatol. Immunol. Res..

[97] Dara A., Vlachogiannis N.I., Fragoulis G.E., Tektonidou M.G., Sfikakis P.P. (2025). In search of biomarkers for prediction of drug treatment responses in rheumatoid arthritis: lessons learned and future perspectives. Autoimmun. Rev..

[98] Gilvaz V.J., Sudheer A., Reginato A.M. (2025). Emerging artificial intelligence innovations in rheumatoid arthritis and challenges to clinical adoption. Curr. Rheumatol. Rep..

[99] Norgeot B., Glicksberg B.S., Trupin L., Lituiev D., Gianfrancesco M., Oskotsky B. (2019). Assessment of a deep learning model based on electronic health record data to forecast clinical outcomes in patients with rheumatoid arthritis. JAMA Netw. Open.

[100] Heard B.J., Rosvold J.M., Fritzler M.J., El-Gabalawy H., Wiley J.P., Krawetz R.J. (2014). A computational method to differentiate normal individuals, osteoarthritis and rheumatoid arthritis patients using serum biomarkers. J. R. Soc. Interf..

[101] Hu C., Dai Z., Xu J., Zhao L., Xu Y., Li M. (2022). Proteome profiling identifies serum biomarkers in rheumatoid arthritis. Front. Immunol..

[102] Chocholova E., Bertok T., Jane E., Lorencova L., Holazova A., Belicka L. (2018). Glycomics meets artificial intelligence–potential of glycan analysis for identification of seropositive and seronegative rheumatoid arthritis patients revealed. Clin. Chim. Acta.

[103] Mannerkorpi M., Gupta S.D., Rieppo L., Saarakkala S. (2025). Diagnostic performance of attenuated total reflectance fourier transform infrared (ATR-FTIR) spectroscopy for detecting osteoarthritis and rheumatoid arthritis from blood serum. Spectrochimica Acta A Mol. Biomol. Spectrosc..

[104] Fukae J., Isobe M., Hattori T., Fujieda Y., Kono M., Abe N. (2020). Convolutional neural network for classification of two-dimensional array images generated from clinical information may support diagnosis of rheumatoid arthritis. Sci. Rep..

[105] Marrero Roche D.E., Chandler K.B. (2024). Clinical glycoprotein mass spectrometry: the future of disease detection and monitoring. J. Mass Spectrom..

[106] Kissel T., Toes R.E., Huizinga T.W., Wuhrer M. (2023). Glycobiology of rheumatic diseases. Nat. Rev. Rheumatol..

[107] He X.P. (2026). Decoding the function of the human glycome using chemical glycoprobes and glycosensor arrays. Chem. Soc. Rev..

[108] Schroeter H.G., Sass S., Heilemann M., Kuner T., Klevanski M. (2026). Nanoscale mapping of the subcellular glycosylation landscape. Adv. Sci..

[109] Bagdonaite I., Malaker S.A., Polasky D.A., Riley N.M., Schjoldager K., Vakhrushev S.Y. (2022). Glycoproteomics. Nat. Rev. Methods Primers.

[110] Dosekova E., Filip J., Bertok T., Both P., Kasak P., Tkac J. (2017). Nanotechnology in glycomics: applications in diagnostics, therapy, imaging, and separation processes. Med. Res. Rev..

[111] Flynn R.A., Pedram K., Malaker S.A., Batista P.J., Smith B.A.H., Johnson A.G. (2021). Small RNAs are modified with N-glycans and displayed on the surface of living cells. Cell.

[112] Sharma S., Jiao X., Yang J., Kwan K.Y., Kiledjian M. (2025). Extracellular exosomal RNAs are glyco-modified. Nat. Cell Biol..

[113] Graziano V.R., Porat J., Ah Kioon M.D., Mejdrová I., Matz A.J., Lebedenko C.G. (2025). RNA N-glycosylation enables immune evasion and homeostatic efferocytosis. Nature.

[114] Li J., Wang L., Deng J., Miao X., Yang X., Zhou Y. (2025). O-Glycosylated RNA identification and prediction by solid-phase chemoenzymatic TnORNA method and PONglyRNA tool. Anal. Chem..

[115] Tkac J., Bertok T. (2023). How glycomic studies can impact on prostate cancer. Expert Rev. Proteomics.

[116] Kellman B.P., Lewis N.E. (2021). Big-Data glycomics: tools to connect glycan biosynthesis to extracellular communication. Trends Biochem. Sci..

[117] Wang Y., Liu Y., Liu S., Cheng L., Liu X. (2024). Recent advances in N-glycan biomarker discovery among human diseases: N-glycan biomarkers in diseases. Acta Biochim. Biophys. Sin..

[118] Tian Y., Wang Y., Zhang Y., Guo J., Zhang P., Li X. (2025). One-step labeling strategy for the profiling of multiple types of protein glycosylation. Anal. Chem..

[119] van Tol B.D.M., Wasynczuk A.M., Gijze S., Mayboroda O.A., Nouta J., Dolhain R.J.E.M. (2025). Comprehensive immunoglobulin G, A, and M glycopeptide profiling for large-scale biomedical research. Mol. Cell Proteomics.

[120] Momčilović A., de Haan N., Hipgrave Ederveen A.L., Bondt A., Koeleman C.A.M., Falck D. (2020). Simultaneous immunoglobulin A and G glycopeptide profiling for high-throughput applications. Anal. Chem..

[121] Trbojević-Akmačić I., Lageveen-Kammeijer G.S., Heijs B., Petrovic T., Deris H., Wuhrer M. (2022). High-throughput glycomic methods. Chem. Rev..

[122] Li Y., Reusch S., van Tol B.D.M., Di Marco F., Wasynczuk A.M., Gijze S. (2026). Fc profiling of polyclonal IgG, IgA and IgM by light chain capturing coupled with NanoRP-LC-MS. JACS Au.

[123] Cheng Y.-H., Lee C.-H., Wang S.-Y., Chou C.-Y., Yang Y.-J., Kao C.-C. (2024). Multiplexed antibody glycosylation profiling using dual enzyme digestion and liquid chromatography-triple Quadrupole mass spectrometry method. Mol. Cell Proteomics.

[124] Reiding K.R., Bondt A., Hennig R., Gardner R.A., O'Flaherty R., Trbojević-Akmačić I. (2019). High-throughput serum N-glycomics: method comparison and application to study rheumatoid arthritis and pregnancy-associated changes. Mol. Cell Proteomics.

[125] Hu Z., Cai S., Tu C., Liu S., Liu X., Dong L. (2025). Serum N-Glycan signatures as potential biomarkers for the detection and monitoring of IgG4-Related disease. J. Proteome Res..

[126] Alvarez M.R.S., Holmes X.A., Oloumi A., Grijaldo-Alvarez S.J., Schindler R., Zhou Q. (2025). Integration of RNAseq transcriptomics and N-glycomics reveal biosynthetic pathways and predict structure-specific N-glycan expression. Chem. Sci..

[127] Munkley J., Elliott D.J. (2016). Hallmarks of glycosylation in cancer. Oncotarget.

[128] Parekh R.B., Dwek R.A., Sutton B.J., Fernandes D.L., Leung A., Stanworth D. (1985). Association of rheumatoid arthritis and primary osteoarthritis with changes in the glycosylation pattern of total serum IgG. Nature.

[129] Youings A., Chang S.C., Dwek R.A., Scragg I.G. (1996). Site-specific glycosylation of human immunoglobulin G is altered in four rheumatoid arthritis patients. Biochem. J..

[130] He Y., Aoun M., Xu Z., Holmdahl R. (2024). Shift in perspective: autoimmunity protecting against rheumatoid arthritis. Ann. Rheum. Dis..

[131] Sharapov S., Timoshchuk A., Zaytseva O., Maslov D.E., Soplenkova A., Elgaeva E.E. (2025). A genome-wide association study in 10,000 individuals links plasma N-glycome to liver disease and anti-inflammatory proteins. Nat. Commun..

[132] Albrecht S., Unwin L., Muniyappa M., Rudd P.M. (2014). Glycosylation as a marker for inflammatory arthritis. Cancer Biomarkers.

[133] Onigbinde S., Adeniyi M., Daramola O., Chukwubueze F., Bhuiyan M.M.A.A., Nwaiwu J. (2025). Glycomics in human diseases and its emerging role in biomarker discovery. Biomedicines.

[134] Morishima N., Iwaisako M., Kamada Y., Nakano M., Shiida M., Ono T. (2025). Generation and validation of antibody 42B1 recognizing galactose-deficient IgG for diagnosis of chronic inflammatory diseases. Clin. Chim. Acta.

[135] Thaçi K., Anthony R.M. (2025). The importance of IgG N-glycosylation in health, disease, and neonatal hemochromatosis. Glycosci. Ther..

[136] Amez Martín M., Wuhrer M., Falck D. (2021). Serum and plasma immunoglobulin G Fc N-glycosylation is stable during storage. J. Proteome Res..

[137] Wu Y., Zhang Z., Chen L., Sun S. (2024). Immunoglobulin G glycosylation and its alterations in aging-related diseases: IgG glycosylation with aging-related diseases. Acta Biochim. Biophys. Sin..

[138] Krištić J., Lauc G. (2024). The importance of IgG glycosylation—What did we learn after analyzing over 100,000 individuals. Immunol. Rev..

[139] Biersteker R., Larsen O.F., Wuhrer M., Huizinga T.W.J., Toes R.E.M., Hafkenscheid L. (2025). Variable domain glycosylation as a marker and modulator of immune responses: insights into autoimmunity and B-cell malignancies. Semin. Immunol..

[140] de Haan N., Falck D., Wuhrer M. (2020). Monitoring of immunoglobulin N- and O-glycosylation in health and disease. Glycobiology.

[141] Shkunnikova S., Mijakovac A., Sironic L., Hanic M., Lauc G., Kavur M.M. (2023). IgG glycans in health and disease: prediction, intervention, prognosis, and therapy. Biotechnol. Adv..

[142] Rudd P.M., Elliott T., Cresswell P., Wilson I.A., Dwek R.A. (2001). Glycosylation and the immune system. Science.

[143] Rombouts Y., Willemze A., van Beers J.J., Shi J., Kerkman P.F., van Toorn L. (2016). Extensive glycosylation of ACPA-IgG variable domains modulates binding to citrullinated antigens in rheumatoid arthritis. Ann. Rheum. Dis..

[144] Nimmerjahn F., Vidarsson G., Cragg M.S. (2023). Effect of posttranslational modifications and subclass on IgG activity: from immunity to immunotherapy. Nat. Immunol..

[145] Kissel T., Ge C., Hafkenscheid L., Kwekkeboom J.C., Slot L.M., Cavallari M. (2022). Surface Ig variable domain glycosylation affects autoantigen binding and acts as threshold for human autoreactive B cell activation. Sci. Adv..

[146] Hafkenscheid L., Bondt A., Scherer H.U., Huizinga T.W.J., Wuhrer M., Toes R.E.M. (2017). Structural analysis of variable domain glycosylation of anti-citrullinated protein antibodies in rheumatoid arthritis reveals the presence of highly sialylated glycans ∗. Mol. Cell Proteomics.

[147] Koers J., Sciarrillo R., Derksen N.I., Vletter E.M., Fillié-Grijpma Y.E., Raveling-Eelsing E. (2023). Differences in IgG autoantibody fab glycosylation across autoimmune diseases. J. Allergy Clin. Immunol..

[148] Peng X., Li Y., Sun X., Ren G., Li H., Wang X. (2025). Therapeutic effect of galactosyltransferase-and sialyltransferase-encoding mRNA in rheumatoid arthritis. Mol. Ther..

[149] Stork E.M., van Rijswijck D.M., van Schie K.A., Hoek M., Kissel T., Scherer H.U. (2024). Antigen-specific fab profiling achieves molecular-resolution analysis of human autoantibody repertoires in rheumatoid arthritis. Nat. Commun..

[150] He Y., Ge C., Moreno-Giró À., Xu B., Beusch C.M., Sandor K. (2023). A subset of antibodies targeting citrullinated proteins confers protection from rheumatoid arthritis. Nat. Commun..

[151] Xu P.C., Gou S.J., Yang X.W., Cui Z., Jia X.Y., Chen M. (2012). Influence of variable domain glycosylation on anti-neutrophil cytoplasmic autoantibodies and anti-glomerular basement membrane autoantibodies. BMC Immunol..

[152] Plomp R., Dekkers G., Rombouts Y., Visser R., Koeleman C.A.M., Kammeijer G.S.M. (2015). Hinge-region O-Glycosylation of human immunoglobulin G3 (IgG3). Mol. Cell Proteomics.

[153] Sołkiewicz K., Kacperczyk M., Krotkiewski H., Jędryka M., Kratz E.M. (2022). O-glycosylation changes in serum immunoglobulin G are associated with inflammation development in advanced endometriosis. Int. J. Mol. Sci..

[154] Kissel T., Derksen V.F., Bentlage A.E., Koeleman C., Hafkenscheid L., van der Woude D. (2024). N-linked Fc glycosylation is not required for IgG-B-cell receptor function in a GC-derived B-cell line. Nat. Commun..

[155] Ding L., Chen X., Cheng H., Zhang T., Li Z. (2022). Advances in IgA glycosylation and its correlation with diseases. Front. Chem..

[156] Stamellou E., Seikrit C., Tang S.C.W., Boor P., Tesař V., Floege J. (2023). IgA nephropathy. Nat. Rev. Dis. Primers.

[157] Falck D., Sokolova M.V., Koeleman C.A., Irumva V., Kirchner P., Schulz S.R. (2025). IgA displays site-and subclass-specific glycoform differences despite equal glycoenzyme expression. Cell Commun. Signal..

[158] Steffen U., Koeleman C.A., Sokolova M.V., Bang H., Kleyer A., Rech J. (2020). IgA subclasses have different effector functions associated with distinct glycosylation profiles. Nat. Commun..

[159] Bondt A., Nicolardi S., Jansen B.C., Kuijper T.M., Hazes J.M., Van Der Burgt Y.E. (2017). IgA N-and O-glycosylation profiling reveals no association with the pregnancy-related improvement in rheumatoid arthritis. Arthritis Res. Ther..

[160] Sebastian A., Alzain M.A., Asweto C.O., Song H., Cui L., Yu X. (2016). Glycan biomarkers for rheumatoid arthritis and its remission status in Han Chinese patients. OMICS.

[161] Wang J.R., Gao W.N., Grimm R., Jiang S., Liang Y., Ye H. (2017). A method to identify trace sulfated IgG N-glycans as biomarkers for rheumatoid arthritis. Nat. Commun..

[162] Sun D., Hu F., Gao H., Song Z., Xie W., Wang P. (2019). Distribution of abnormal IgG glycosylation patterns from rheumatoid arthritis and osteoarthritis patients by MALDI-TOF-MSn. Analyst.

[163] Mayboroda O.A., Lageveen-Kammeijer G.S.M., Wuhrer M., Dolhain R.J.E.M. (2023). An integrated glycosylation signature of rheumatoid arthritis. Biomolecules.

[164] Wang Y., Lai Y., Wang J., Wu J., Yu H., Xiao Y. (2025). Serum IgG N-glycans act as serum biomarkers for differentiation of cold and heat pattern in rheumatoid arthritis. Chin. Med..

[165] Tsezou K.I., Ghorasaini M., Verhoeven A., Iliou A., Benaki D., Vlachoyiannopoulos P.G. (2025). Comprehensive lipoprotein and glycoprotein characterization in rheumatoid arthritis plasma and association with clinical markers. Front. Mol. Biosci..

[166] Embade N., Millet O. (2025). NMR-based quantification of GlycA and GlycB: advancing inflammatory biomarkers in clinical diagnostics. Methods Mol. Biol..

[167] Llop D., Paredes S., Rosales R., Amigó N., Masana L., Ribalta J. (2024). Comprehensive analysis of glycoprotein profiles and their association with cardiovascular disease-related microRNAs in rheumatoid arthritis, metabolic disorders, and controls. Sci. Rep..

[168] Reiding K.R., Vreeker G.C., Bondt A., Bladergroen M.R., Hazes J.M., Van der Burgt Y.E. (2018). Serum protein N-glycosylation changes with rheumatoid arthritis disease activity during and after pregnancy. Front. Med..

[169] Szabó D., Gyebrovszki B., Szarka E., Auer F., Rojkovich B., Nagy G. (2025). Immunoglobulin G subclass-specific glycosylation changes in rheumatoid arthritis. Int. J. Mol. Sci..

[170] Ma W., Xu Z., Jiang Y., Liu J., Xu D., Huang W. (2023). Divergent enzymatic assembly of a comprehensive 64-Membered IgG N-glycan library for functional glycomics. Adv. Sci..

[171] Magorivska I., Döncző B., Dumych T., Karmash A., Boichuk M., Hychka K. (2018). Glycosylation of random IgG distinguishes seropositive and seronegative rheumatoid arthritis. Autoimmunity.

[172] Huang C., Liu Y., Wu H., Sun D., Li Y. (2017). Characterization of IgG glycosylation in rheumatoid arthritis patients by MALDI-TOF-MSn and capillary electrophoresis. Anal. Bioanal. Chem..

[173] Su Z., Xie Q., Wang Y., Li Y. (2020). Abberant immunoglobulin G glycosylation in rheumatoid arthritis by LTQ-ESI-MS. Int. J. Mol. Sci..

[174] Yang H., Lin Z., Wu B., Xu J., Tao S.-C., Zhou S. (2024). Deciphering disease through glycan codes: leveraging lectin microarrays for clinical insights: deciphering disease through glycan codes. Acta Biochim. Biophys. Sin..

[175] Valk A.M., Koers J., Derksen N.I.L., Hogenboom L., van Kempen Z., Killestein J. (2025). Elevated Fab glycosylation of autoantibodies maintained during B cell depletion therapy. Sci. Rep..

[176] Stümer J., Biermann M.H.C., Knopf J., Magorivska I., Kastbom A., Svärd A. (2017). Altered glycan accessibility on native immunoglobulin G complexes in early rheumatoid arthritis and its changes during therapy. Clin. Exp. Immunol..

[177] Kratz E.M., Kacperczyk M., Kokot I., Piwowar A., Konopska B., Sokolik R. (2024). Glycosylation pattern of serum clusterin in psoriatic arthritis and rheumatoid arthritis—the search for new diagnostic glycomarkers. Int. J. Mol. Sci..

[178] Takeshita M., Kuno A., Suzuki K., Matsuda A., Shimazaki H., Nakagawa T. (2016). Alteration of matrix metalloproteinase-3 O-glycan structure as a biomarker for disease activity of rheumatoid arthritis. Arthritis Res. Ther..

[179] Gińdzieńska-Sieśkiewicz E., Radziejewska I., Domysławska I., Klimiuk P.A., Sulik A., Rojewska J. (2016). Changes of glycosylation of IgG in rheumatoid arthritis patients treated with methotrexate. Adv. Med. Sci..

[180] Falck D., Wuhrer M. (2024). GlYcoLISA: antigen-specific and subclass-specific IgG Fc glycosylation analysis based on an immunosorbent assay with an LC–MS readout. Nat. Protoc..

[181] Xu Z., Liu Y., He S., Sun R., Zhu C., Li S. (2023). Integrative proteomics and N-glycoproteomics analyses of rheumatoid arthritis synovium reveal immune-associated glycopeptides. Mol. Cell Proteomics.

